# Comparative analysis of traditional and smart antibacterial textiles for wearable technology; a comprehensive review

**DOI:** 10.1039/d6ra01779g

**Published:** 2026-09-01

**Authors:** Azam Ali, Muhammad Zaman Khan, Jiri Militký, Jakub Wiener, Tazeen Rana, Eman Kashita

**Affiliations:** a Department of Material Sciences, Technical University of Liberec 460 15 Czech Republic azam.ali@tul.cz muhammad.zaman.khan@tul.cz jiri.militky@tul.cz Jakub.wiener@tul.cz; b Department of Physics, College of Science, Qassim University Buraydah 51452 Qassim Saudi Arabia t.ull@qu.edu.sa E.ELSAYED@qu.edu.sa

## Abstract

The growing use of wearable technologies in healthcare, athletics, and defense has created a strong demand for textile materials that are durable, safe, and capable of providing long-term antimicrobial protection. Conventional antibacterial fabrics typically depend on passive agents such as metal ions and synthetic biocides. Although effective initially, these systems often suffer from rapid activity loss due to uncontrolled release, limited wash durability, and concerns related to environmental burden and potential toxicity. To address these limitations, next-generation smart antibacterial textiles have been developed using stimuli-responsive polymers, nanofibers, hydrogels, and conductive materials that enable on-demand antimicrobial activity in response to pH, moisture, heat, or mechanical strain. This review compares conventional and smart antibacterial textiles in terms of activation strategy, working mechanism, durability, fabrication routes, antibacterial performance, and sustainability. Traditional approaches, including pad-dry-cure and sol–gel methods, can provide effective antimicrobial action but are often constrained by poor wash fastness and uncontrolled leaching of active agents. In contrast, smart systems fabricated through electrospinning, microencapsulation, or conductive coating generally offer improved durability, adaptive response, and reduced environmental impact. A key advantage of smart textile design lies in the combined engineering of the textile matrix and the incorporation of stimuli-responsive components, enabling multifunctional materials with intelligent behavior toward external triggers. These systems also support integration with sensing and energy-harvesting functions, which is essential for advanced wearable platforms. However, challenges remain in large-scale reproducibility, cytotoxicity assessment, and the lack of standardized protocols for evaluating dynamic antibacterial performance. Overall, this review provides a comparative framework that highlights the shift from passive antibacterial coatings toward multifunctional, responsive textiles for next-generation wearable applications.

## Abstract 2

The growing use of wearable technologies in healthcare, athletics, and defense has created a strong demand for textile materials that are durable, safe, and capable of providing long-term antimicrobial protection.

## Graphical abstract

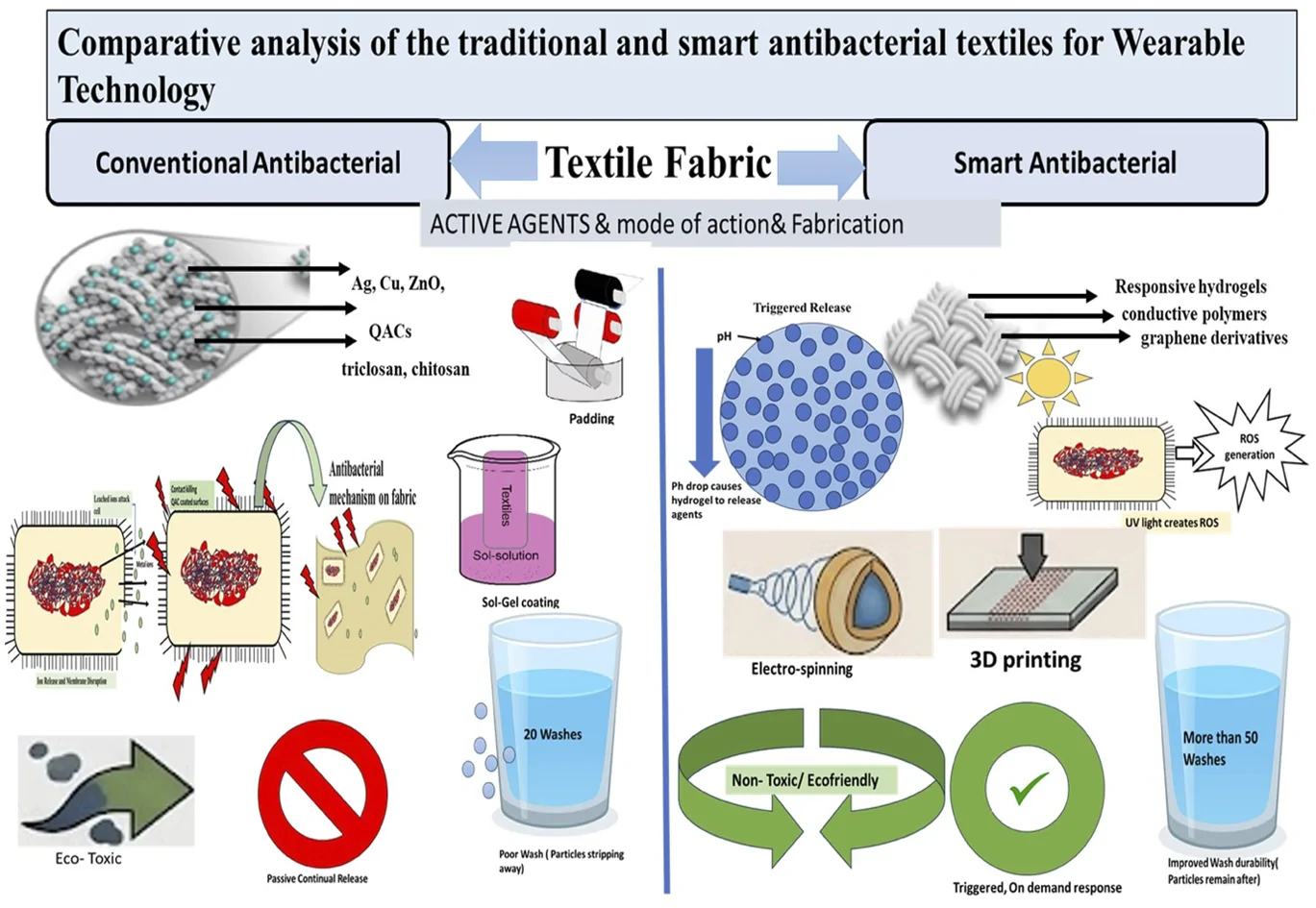

## 1 Introduction

Over the past several decades, demand for antibacterial textiles has increased substantially, particularly in healthcare, where these materials are used to reduce pathogen transmission during surgical procedures and post-operative care.^1^ By inhibiting microbial colonization, antibacterial finishes not only improve hygiene but also prolong textile service life by minimizing fiber degradation, discoloration, and deterioration caused by microbial growth.^2^ More recently, advances in wearable technologies, flexible electronics, and functional materials have transformed conventional textiles into multifunctional platforms capable of sensing, communication, therapeutic delivery, and physiological monitoring.^3,4^ Consequently, antibacterial textiles are evolving from passive protective materials into intelligent systems that simultaneously provide antimicrobial protection and advanced wearable functionalities. Even though these advances, conventional textile substrates remain highly susceptible to bacterial contamination because they readily absorb sweat, sebum, and other organic residues that promote microbial adhesion, proliferation, unpleasant odors, discoloration, and infection. To mitigate these problems, antibacterial textiles have traditionally been fabricated by incorporating inorganic or organic antimicrobial agents through coating, padding, immersion, or finishing processes. However, these approaches often exhibit limited washing durability because gradual leaching of the active agents reduces long-term antimicrobial efficacy while raising potential toxicity and environmental concerns. Furthermore, continuous release of antimicrobial agents, irrespective of microbial challenge, may disturb the natural skin microbiome and contribute to the emergence of antimicrobial resistance. These shortcomings become increasingly significant as antibacterial textiles are integrated with wearable electronic devices, where long-term functionality, flexibility, durability, and biocompatibility are essential.^5^

To overcome these limitations, considerable research has focused on the development of smart antibacterial textiles incorporating stimuli-responsive materials, including conductive polymers, graphene-based nanomaterials, hydrogels, microcapsules, and multifunctional nanocomposites. Unlike conventional antibacterial finishes, these systems respond to external or physiological stimuli, such as pH, temperature, moisture, mechanical deformation, or electrical signals, enabling controlled activation of antimicrobial functions only when required. This on-demand response improves antimicrobial efficiency while minimizing unnecessary biocide release, reducing environmental impact, and enhancing wearer safety. In biomedical applications, stimuli-responsive antibacterial systems also enable localized delivery of therapeutic agents, improving bacterial eradication, reducing systemic toxicity, and enhancing treatment of biofilm-associated infections surrounding implants and wound dressings. Recent advances in nanomedicine have further accelerated the development of such intelligent antimicrobial platforms by improving controlled release behavior, reducing carrier leakage, and enhancing therapeutic efficacy against drug-resistant pathogens.^6^ The rapid development of smart antibacterial materials has also expanded the functionality of wearable textiles beyond infection control. Modern antibacterial textile systems can integrate sensing, data communication, thermal regulation, self-cleaning, ultraviolet protection, and physiological monitoring within a single flexible platform, making them increasingly attractive for healthcare, sportswear, military, and personal protective application. These developments have stimulated growing academic interest and commercial investment in multifunctional antibacterial wearable technologies.^7^

This review provides a comprehensive comparison of conventional and smart antibacterial textiles by critically examining their antimicrobial agents, fabrication strategies, mechanisms of antibacterial action, functional performance, and application potential. Particular emphasis is placed on recent advances in stimuli-responsive antibacterial systems based on hydrogels, conductive polymers, graphene derivatives, microcapsules, and nanofibrous structures, highlighting their ability to combine controlled antimicrobial activity with wearable electronic functionality and enhanced user comfort.^8^ In addition, the review discusses the major challenges associated with durability, large-scale manufacturing, environmental safety, and long-term performance.

Despite substantial progress, several important research gaps remain. Most published studies primarily evaluate antibacterial efficacy while providing limited systematic comparison between conventional and smart antibacterial textiles under realistic service conditions. Critical aspects, including physiological responsiveness, washing durability, microbiome compatibility, environmental leaching, and long-term functional stability, remain insufficiently investigated. Moreover, the absence of standardized evaluation protocols for stimuli-responsive antibacterial performance limits meaningful comparison across different studies, while the integration of antibacterial materials with conductive networks, flexible sensors, and energy-harvesting systems remains at an early stage of development. By systematically comparing conventional and smart antibacterial textile technologies, this review identifies current limitations, highlights emerging opportunities, and outlines future research directions toward the development of durable, environmentally sustainable, and multifunctional antibacterial wearable systems.

## 2 Antibacterial textiles

Textiles used in daily and clinical environments can easily become a great source of diverse microorganisms, making a potential route for cross infection. Even though routine laundering is the most effective means of reducing microbial load, it is often impractical larger scale settings such as hospitals, where garments and linens are continuously reused. These limitations have driven the development of antibacterial textiles designed to inhibit microbial survival and also to reduce the risk of textile mediated transmission.^9^ Antibacterial fabrics are specially designed fabrics to reduce or eliminate the load of microorganisms such as fungi, bacteria, and viruses on the outer surfaces of fabrics through physical, chemical, or biological mechanisms. These protective strategies typically include the embedding antimicrobial agents such as metal nanoparticles, chitosan, or quaternary ammonium compounds within the textile matrix, modifying fiber surfaces to generate bactericidal interfaces, or the integration of bioactive molecules capable of disrupting microbial metabolism. Such textiles have significant role in enhancing hygiene, preventing infections, and extending fabric durability, particularly in healthcare, sportswear, and smart wearable applications.^10^ Antibacterial qualities must coexist with flexibility, electrical conductivity, and skin compatibility in the context of wearable technology. Such multifunctional designs offer great potential for smart, protective, and healthcare fabric applications by improving the washing durability, electrical stability, and antibacterial effectiveness of conductive textiles.^11^ Antibacterial textiles can be broadly classified into conventional and smart systems as describe in Table 1.

**Table 1:** Comparative analysis of conventional and smart antibacterial textiles

Textile type	Functional principle	Common antibacterial agents	Activation behavior	Fabrication approaches	Main advantages	Key limitations	Ref.
Conventional antibacterial textiles	Passive antibacterial activity through continuous ion release or contact killing	Ag, Cu, ZnO, QACs, triclosan, chitosan	Continuous and non-responsive	Pad-dry-cure, dip coating, sol–gel, exhaust, electroless plating	Simple processing, rapid antibacterial action, industrial scalability	Uncontrolled leaching, reduced wash durability, possible cytotoxicity	10 and 11
							12 and 13
Smart antibacterial textiles	Stimuli-responsive or self-regulated antibacterial activity	Responsive hydrogels, microcapsules, conductive polymers, graphene derivatives, photocatalytic nanocomposites	Triggered by pH, moisture, light, temperature, strain, or electrical stimuli	Electrospinning, microencapsulation, plasma functionalization, layer-by-layer assembly, hybrid deposition	Controlled release, multifunctionality, improved durability, wearable compatibility	Complex fabrication, higher cost, lack of standardized evaluation methods	5, 12, 14 and 15

## 3 Conventional antibacterial fabrics

Conventional antibacterial fabrics treated with or made from materials that inhibit or kill every kind of microorganisms. These fabrics are widely used in healthcare, sportswear, military uniforms, and household textiles to prevent odor, infection, and degradation caused by microbial growth. Traditional fibers such as nylon, cotton, polyester or their blends, have been achieved antibacterial properties by applying coatings and chemical finishes. The widely used antibacterial agents for this purpose are chitosan, QACs (Quaternary Ammonium Compounds), triclosan and metal-based compounds (such as copper, zinc and silver). Across these all, silver–metal compounds, are found to be predominant due to their compatibility, durability as well as intense broad-spectrum antimicrobial activity with a number of Textile surfaces.^12^ However, a number of challenges are being faced due to these traditional antibacterial treatments. The potential barrier is the leaching out of metal–nanoparticles and environmental pollution. Likewise, some factors related to skin sensitivity, ecological disruption and microbial resistance are raised by QACs and triclosan. Traditional fabrics having antibacterial properties are still being used because of their competitive pricing, efficacy and convenient usage in previously existing fabric manufacturing processes.^7^

### 3.1 Mechanisms of action

The antibacterial activity of conventional textiles typically relies on one or more of the following mechanisms.

#### 3.1.1 Ion release (leaching mechanism)

Nanoparticles of metal and their oxides, such as TiO2, Ag, Cu, and ZnO exhibit antibacterial action primarily by releasing metal ions that interact with and damage bacterial membranes and internal cell components. In fact, these ions interact with thiol, carboxyl, and phosphate groups present in proteins and DNA, resulting in membrane disruption, oxidative imbalance, enzyme inactivation, and DNA impairment. The sustained release of ions generates reactive oxygen species (ROS) which further amplifies intracellular damage cellular damage and metabolic imbalance, ultimately leading to bacterial death. While this mechanism offers strong and broad-spectrum antimicrobial action, excessive ion release can lead to cytotoxicity and reduced material durability, highlighting the need for controlled-release systems in textile applications.^16^

#### 3.1.2 Contact killing

Surfaces coatings with quaternary ammonium compounds (QACs), *N*-halamines,^17^ or cationic polymers kill bacteria through electrostatic attraction by interacting with negatively charged bacterial membranes. This non-leaching technique (relies only on contact) are less less effective on rough or contaminated surfaces (due to bumps or improper contact).^18^ The *N*-halamine coated fabrics totally relies on contact based antibacterial activity through by transferring oxidative halogens to microbes and leads to membrane disruption. As their halogen groups are able to regenerate; which enable long lasting, non-leaching antimicrobial effectiveness. Current review also focused on improving their structural stability and compatibility with textile fiber.^17^ A study confirms that polymer surfaces functionalized with quaternary ammonium compounds (QACs) exhibit contact-based antibacterial activity by disrupting negatively charged bacterial membranes, leading to cell lysis. Their non-leaching, durable action makes them ideal for long-term antimicrobial textiles. However, optimizing biocompatibility and structural design remains essential to enhance safety and efficacy.^19^

#### 3.1.3 2Reactive oxygen species (ROS) generation

Reactive oxygen species (ROS) generation is one of the most widely recognized response underlying metal ion mediated antibacterial activity. The formation of these species is due to the incomplete or intermediate oxidation reaction for the molecules of oxygen. The term ROS also encompasses non-radical oxygen-based oxidants, such as hydrogen peroxide (H_2_O_2_). Nevertheless, aerobic respiration inherently produces reduced oxygen species which include H_2_O_2_ and superoxide (O_2_˙^−^), which may further react with intracellular iron, promoting auto oxidation processes.^20^ However, when the equilibrium between ROS generation and antioxidant defenses is disrupted, ROS accumulate and inflict extensive oxidative damage on proteins, nucleic acids, and membrane lipids, ultimately driving bacterial cell death.^21,22^ Several studies observed that both essential ions (*e.g.* Fe^2+^, Cu^2+^) and non-essential toxic metals (*e.g.* Cr^6+^, As^3+^) ions can elevate the intracellular ROS production. Thus, a substantial component of metal-induced toxicity is attributed to this ROS surge and the resulting oxidative injury to vital cellular components.^23^ Photocatalytic agents produce ROS under light exposure, oxidizing microbial membranes. Although efficient, these systems depend on illumination intensity and can degrade textile polymers under prolonged exposure.^24^ Reactive oxygen species involved in antibacterial activity may arise through two distinct pathways. In ion-mediated (“dark ROS”) systems, transition metal ions such as Ag^+^, Cu^2+^, and Fe^2+^ catalyze intracellular oxidative reactions even in the absence of light, leading to membrane oxidation, protein damage, and DNA disruption. In contrast, photocatalytic ROS generation occurs in photoactive materials such as TiO_2_ and ZnO under light irradiation, where electron–hole pair formation produces hydroxyl radicals and superoxide species capable of oxidizing bacterial components. Distinguishing these mechanisms is important because photocatalytic systems depend strongly on illumination conditions, whereas ion-mediated ROS activity can occur continuously under physiological environments.^25^

#### 3.1.4 Physical barrier or entrapment

Chitosan based coatings function as physical barriers that hinder microbial adhesion and proliferation on fabric surfaces. Acting as a semi-permeable film, chitosan forms a protective layer that entraps or isolates bacteria, preventing their direct contact with the textile substrate. In addition, its polymeric network limits moisture and nutrient diffusion, creating unfavorable conditions for microbial growth. This mechanism is particularly valuable for hygienic and packaging textiles where non-leaching, protective finishes are preferred.^26^ A major limitation across these mechanisms is non-selective activity agents attack both harmful and beneficial microflora and loss of potency after washing or abrasion.^27^

### 3.2 Conventional agents for traditional textiles

Antimicrobial finishes applied to textiles are commonly classified into three broad categories based on their chemical composition and mechanism of action: (i) inorganic agents, (ii) natural agents, and (iii) synthetic agents.^28,29^ Natural antimicrobial agents are primarily derived from plant or animal sources. Plant-based biopolymers and extracts, in particular, have attracted attention as environmentally friendly alternatives due to their biodegradability and relatively low toxicity. Although their antimicrobial activity may be milder compared to metal-based systems, they offer advantages in terms of sustainability and biocompatibility. Synthetic antibacterial agents, including compounds such as triclosan and quaternary ammonium-based materials (*e.g.*, synorin chloride), are engineered to deliver strong and durable antimicrobial performance,^30^ These systems often involve functional polymers or nanoparticle-based coatings designed to withstand repeated laundering and mechanical stress. Incorporating such agents into textile matrices enhances microbial resistance while preserving fabric flexibility and mechanical integrity.^31^

#### 3.2.1 Natural agents for antibacterial textile

Natural antimicrobial compounds obtained from plant and animal sources are increasingly recognized as promising substitutes for conventional synthetic agents in textile applications. Their growing interest stems from inherent advantages such as biodegradability, favorable biocompatibility, and activity against a wide range of microorganisms, making them attractive options for more sustainable fabric treatments.

#### 3.2.2 4Plant-derived compounds

Plant-derived agents including essential oils, flavonoids, tannins, alkaloids, and polyphenols show antibacterial potential by disrupting metabolic process.^29^ Some plant exhibit notable antimicrobial performance, with neem-based treatments yielding about bacterial reductions of 59–69% against Gram-negative and 58–64% against Gram-positive bacteria.^32,33^ Pomegranate peels retain high levels of tannins agent, ellagic acid, and punicalagin which demonstrate very effective antibacterial activity with up to an 80% bacterial reduction in wool fabrics (coated with Pomegranate peels extract).^34,35^ Moreover, some other natural antipathogenic products like honey and oils exert antimicrobial effects by creating osmotic imbalance, lowering pH to acidic side and by generating reactive oxygen species.^36,37^ Herbal and polyphenol-based systems, such as alfalfa and EGCG-loaded hydrogels, have also been explored for wound-healing textiles due to their combined antibacterial and antioxidant effects that promote tissue regeneration.^38,39^

#### 3.2.3 Animal derived antibacterial

Antipathogenic agents sourced from animals most notably chitosan and sericin offer an excellent biocompatibility and environmental safety for textile finishing applications. Chitosan, obtained by the process of deacetylation (of chitin found in crustacean shells), exhibits inherent antibacterial effects and can be incorporated through coating, impregnation, or layer-by-layer assembly. For example, a research work showed, a chitosan/carboxymethylcellulose composite coating over textile surface showed 99.99% antiviral effectiveness against coronavirus.^40^ Regardless of their promising potential, challenges remain in improving the stability, controlled release, and wash durability of natural antibacterial agents in textile applications.^41^

#### 3.2.4 Metal and metal-oxide nanoparticles

Metal and metal-oxide nanoparticles as antibacterial agents are commonly used over the textile surface or even inside structure of fabrics due to their long-lasting stability, and resistance to degradation. They are able to deliver sustain release (ions or agents) typically involves disrupting bacterial membranes, hindering metabolic process, or generating reactive oxygen species (ROS), providing broad-spectrum antimicrobial efficacy.^28^ Silver (Ag) nanoparticles are the most extensively studied inorganic antimicrobials for textiles, exhibiting strong antibacterial, antifungal, and antiviral properties through the controlled release of Ag^+^ ions and nanoparticles.^42–44^ Their efficacy depends on particle size, shape, and concentration, with smaller Ag NPs showing enhanced ion release and microbial interaction.^45^ Nanoparticle based materials for surface functionalization is widely used to impart antimicrobial performance to both natural and synthetic fabrics. Silver nanoparticles, range up to 10 nm, are among the most effective due to their broad spectrum's toxicity toward microbes, comparatively low cytotoxicity to human cells, and high durability on fibers. Their use also enhances dyeability and adds self-cleaning and antiviral functionality, including reported inhibition of SARS-CoV-2.^46^ A copper ion-impregnated textile (Cu-IT) was developed by strongly coordinating copper ions within the cellulose matrix of cotton fibers, overcoming the leaching limitations of conventional surface-coated antimicrobial textiles. The Cu-IT exhibited excellent antiviral and antibacterial activity against multiple pathogens, including influenza A virus, *E. coli*, *Pseudomonas aeruginosa*, and *Bacillus subtilis*, due to the intrinsic antimicrobial properties of copper. Additionally, the textile demonstrated enhanced mechanical durability, water/air retainability, wash resistance, cost-effectiveness, eco-friendliness, and scalability for potential applications in medical, household, and public environments.^36^ Other metal or metal oxide nanostructures such as CuO, TiO_2_, ZnO, Au, and Sn-based systems rely on multiple mechanisms. For instance, CuO-coated fabrics exhibit antibacterial action through copper ion release, membrane contact, and ROS generation, while amino-functionalized TiO_2_ nanoparticles deposited *via* sol–gel and hydrothermal methods have shown strong inhibition against *E. coli* and *S. aureus* on cotton substrates.^47^

#### 3.2.5 Carbon-based materials

Carbon nanotubes (CNTs), particularly single-walled CNTs (SWCNTs), exhibit excellent antibacterial characteristics, largely attributed to their tubular structure, greater surface area, and and capacity to mechanically compromise bacterial cell membranes.^48–50^ CNT-coated fabrics as well as ZnO-BTCA-coated fabrics prepared through exhaustion or composite coating methods, demonstrate enhanced and durable antibacterial performance toward both Gram-positive and Gram-negative microorganisms, along with improved electrical conductivity.^51,52^ Graphene-based materials have shown strong antibacterial performance, making them promising candidates for integration into conventional textile finishes. Graphene, characterized by its sp^2^-hybridized carbon lattice, exhibits antibacterial activity primarily through direct physical interactions with microbial cell membranes. Similar to other carbon-based nanomaterials, including carbon nanotubes (CNTs), graphene can induce membrane stress, leading to structural disruption and leakage of intracellular components.^53^ In a related approach, the prepared TiO_2_/GO/cellulose acetate nanofiber composites using electrospinning and subsequently integrated them onto cotton substrates to produce TiO_2_/GO/CA@cotton. The resulting material exhibited antibacterial efficiencies exceeding 95% against *Bacillus subtilis* and *Bacillus cereus*, while also enhancing surface hydrophilicity,^54^.

#### 3.2.6 Synthetic antimicrobial agents for textile

Among synthetic agents, *N*-halamines (already shortly discussed in aforementioned sections) represent a major class of nitrogen–halogen (N–X) antimicrobials, most commonly based on chlorine-based systems. They act by releasing oxidative halogen species which in turn disrupt the cellular metabolism and leads to bacterial death.^30^ If they are applied through pad-dry process followed by chlorination (to maintain synergistic effect) they used to provide very effective antibacterial efficacy, yields up to 85% activity retention after 15 days. The can easily applied over cellulosic structures (cotton), protein fibers (wool) or even on synthetics such as polyester, and polyamides.^1^ Moreover, their hybrid systems with siloxanes or quaternary ammonium salts further provide the synergic effect and enhance performance and durability, making *N*-halamines especially attractive for medical and hygiene fabrics.^30^ Triclosan (C_12_H_7_Cl_3_O_2_), is another non-ionized chlorinated bisphenol which is mostly applied now a days as a textile finishing. Its antibacterial effect arises by interacting with lipid biosynthesis and it may also disrupt the microbial cell membranes. Due to its washing fastness property, it has been applied over the different kind of fabric over three decades.^1^ For cellulosic fibers, it is commonly applied with polycarboxylic crosslinkers or *via* β-cyclodextrin host guest complexes to improve fixation and sustained release. Encapsulation in biodegradable polylactide has also been reported to enhance long-term stability and environmental compatibility.^1^Table 2 summarizes the major antibacterial agents, their key active components, mechanisms of action, and representative textile applications.

**Table 2:** Classification of major antibacterial agents used in conventional textiles

Category	Source/example	Key active components	Mechanism of action	Applications in textiles	Ref.
Natural (plant-based)	- Polyphenols (tea, coffee, EGCG)	Antioxidants	Antibacterial, anti-inflammatory, angiogenesis	Hydrogel dressings, healing fabrics	55
	- Neem	Azadirachtin (limonoid)	Cell wall breakdown	Antibacterial fabrics	40
	Aloe vera & Myrobalan	Bioactive phytochemicals	Bacterial reduction (59–69%)	Herbal-treated fabrics	32 and 33
	Pomegranate (*Punica granatum*)	Punicalagin, tannins, ellagic acid	Dye + antimicrobial, disrupt metabolism	Antibacterial dyeing textiles	56
	Honey	High sugar, acidic pH, peroxides	Osmotic stress, biofilm inhibition	Wound healing textiles	36 and 37
	Essential oils (Thymol, Carvacrol)	- Phenolic compounds	Membrane disruption, ROS generation	Biopolymer nanofiber wound dressings	55
Natural (animal-based)	Chitosan (from chitin)	- Biopolymer polysaccharide	Cell wall disruption, hydrogels, antiviral	Coatings, wound dressings, medical textiles	40
	Sericin (silkworm cocoon)	- Protein	Biocompatible, antibacterial	Skin-friendly fabrics, wound care	57
	Chitosan derivatives (QAC – nanoparticles)	- Amino groups (–NH_2_)	Electrostatic interaction with bacteria, membrane rupture	Antibacterial finishing, immobilized coatings	58
	Collagen hydrolysate	- Bioactive peptides	Enhances cell adhesion, mild antibacterial effect	Wound care dressings, biomedical fiber	59
	Alginate	Polysaccharide (guluronic/mannuronic acid)	Ion exchange, gel formation, moisture retention	Wound healing, antimicrobial absorbent fibers	60
Inorganic	Silver nanoparticles	Ag^+^, Ag NPs	Disrupt membranes, ROS production	Medical textiles, hospital fabrics	56
	Copper oxide nanoparticles (CuO, Cu_2_O)	Cu^+^, Cu^2+^ ions	Ion release, oxidative stress	Antibacterial multifunctional fabrics	61 and 62
	ZnO NPs	Zn^2+^ ions	ROS generation, membrane	UV-protective antibacterial	63
	TiO_2_ NPs	TiO_2_	Photocatalytic ROS generation	Self-cleaning and antibacterial	64 and 65
	MgO NPs	MgO	Alkalinity-based membrane damage, ROS	Thermally stable antibacterial coatings	57
Carbon based	Carbon nanotubes (CNTs)	SWCNTs, MWCNTs	Physical piercing, adsorption	Antibacterial coatings on cotton	48
	Graphene oxide (GO, RGO)	Graphene sheets	Membrane cutting, ROS, ion implantation	Dip-coated cotton, composites	49 and 50
Synthetic	Silver nanoparticles (AgNPs)	Nano-Ag (∼10 nm)	Antibacterial, antiviral, durable	Self-cleaning fabrics, SARS-CoV-2 protective	65
	*N*-halamines	N–Cl functional groups	Chlorine release, metabolic disruption	Medical fabrics, hygiene products	66
	Triclosan	Chlorinated bisphenol	Inhibits lipid biosynthesis	Polyester, nylon, polypropylene	67
	Polyhexamethylene biguanide	Biguanide polymer	Destabilizes bacterial membranes	Hospital textiles, hygiene products	68
	Biguanidine	Biguanide groups	Membrane disruption, cationic interaction	Antibacterial coatings, reusable fabrics	69

## 4 Fabrication techniques for conventional antibacterial textiles

Antibacterial textiles are engineered using a range of strategies that integrate bioactive agents into or onto fabric substrates to suppress microbial proliferation. These approaches generally fall into chemical, physical, or bio-based categories and employ either surface modification or bulk incorporation techniques. Conventional finishing methods apply antimicrobial agents on the textile, typically with binders or crosslinkers to improve fixation and wash durability. Several fabrication routes are used to incorporate antibacterial agents into textile substrates. The choice depends on fiber type, desired durability, and production cost.^70^

### 4.1 Pad dry cure method

**Fig. 1:**
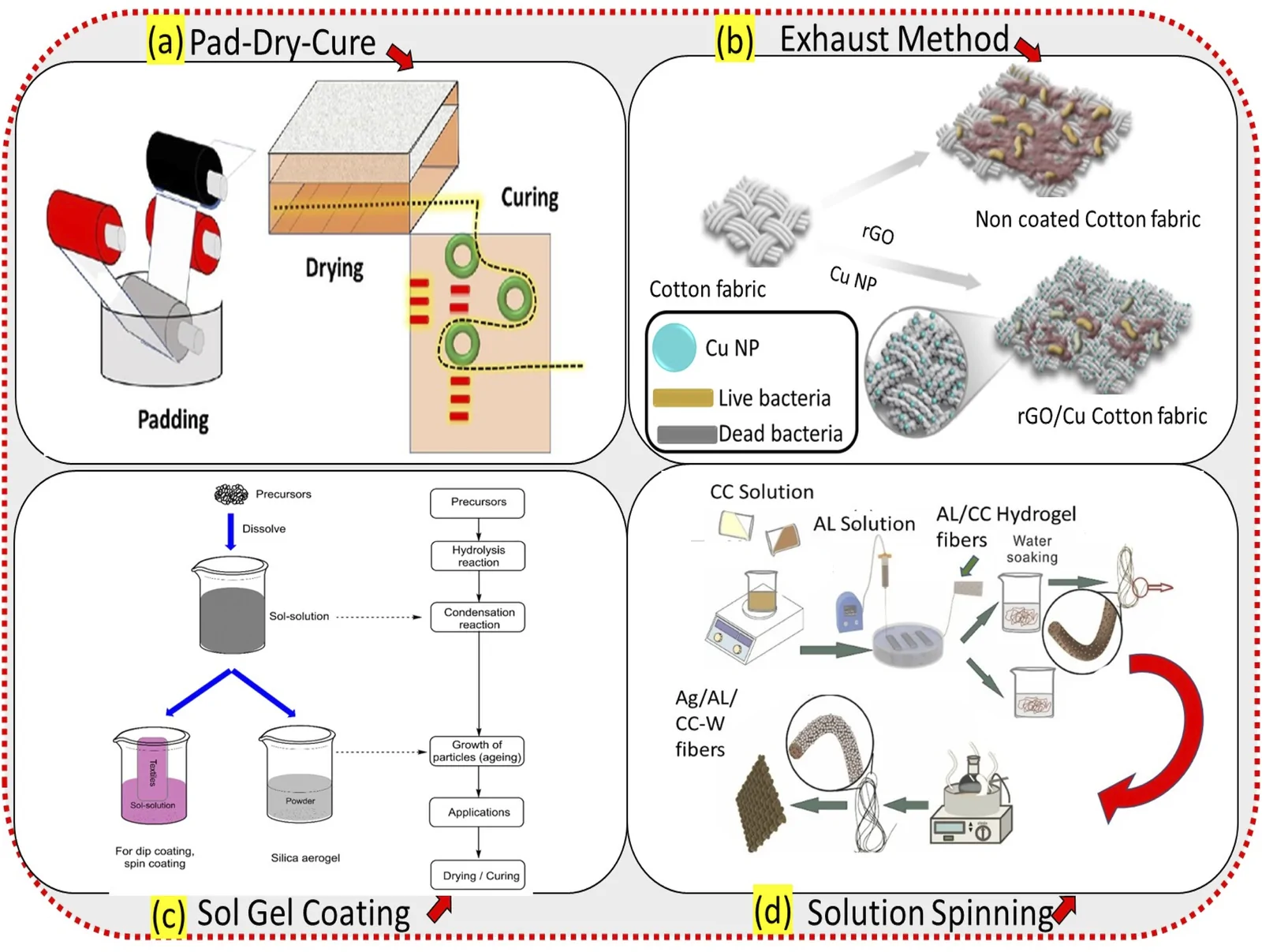
(a) Application of antibacterial finish by pad-dry-cure process.^13^ Reprint with permission from [Shaukat], [green synthesis of antipathogenic particles by utilizing the citrus plant waste and their application in medicated fabrics]; published by [Sage], [2024]. (b) Schematic of preparation of exhaust method to apply antibacterial agents (rGO/Cu) nanocomposites for the prevention of biofilm formation. Redrawn with permission from [Kim], [antibacterial and biofilm-inhibiting cotton fabrics decorated with copper nanoparticles grown on graphene nanosheets]; published by [scientific report], [2023].^83^ (c) Steps involved in a sol–gel process. Redrawn with permission from [A. P. Periyasamy], [progress in sol–gel technology for the coatings of fabrics]; published by [MDPI], [2020].^78^ (d) Schematic illustration of the solution spinning process for the Ag/AL/CC-g fibers.^82^ Redrawn with permission from [Chen], [ultraviolet protection and antibacterial properties of textile fabric made of silver nanoparticles/alkaline lignin/regenerated cellulose fiber], [published by [Elsevier], 2025].^82^

It's a type of chemical finishing method refers to conventional wet-chemical treatments where an antibacterial agent is applied to a completed textile, typically by procedure of drying and heat curing to fix the agent onto the fibers, the process is known as pad dry cure process.^71^ In a process of pad dry, the material is passed through an aqueous medium of the antimicrobial (often with binders or crosslinkers), squeezed through rollers to a controlled wet pick-up, then dried and cured at elevated temperature. This bonds or chemically links the agent to the textile, yielding durable finish.^72^ A recent study applied a plant derived extract, leaves of *Rumex steudelii* Hochst., onto cotton by pad dry cure method. The procedure involves the soaking of fabric for 5 min, passed through a nip and dried at 100 °C, then proceed by curing at 130 °C, with citric acid serving as a crosslinker agent. The fabric made by this procedure produced substantial inhibition zones (30–32 mm) against *E. coli* and *S. aureus*, maintaining its integrity even after several machine washes. The procedure of Pad dry cure is used commonly due to its effectiveness and scalability but susceptible to uneven distribution and reduced activity after repeated washing.^73^ Shaukat *et al.*^13^ tried to apply green synthesized nanoparticles on textile by pad-dry-cure method (Fig. 1(a)). Antipathogenic fabrics were coated with different types of metallic nanoparticles.^13^ The citrus plant waste was collected and processed to extract the bioactive molecules for the green synthesis of metal particles. A green synthesis approach was used to produce highly concentrated and stable colloidal dispersions of silver (Ag-NPs), copper (Cu-NPs), and zinc oxide nanoparticles (ZnO-NPs). The method relied on the self-assembly of their respective metal salts and on agents derived from plants, eliminating the need for any toxic substance or chemical.^13^

### 4.2 Dip coating or exhaust method

Dip or exhaust method is akin to dye bath method in which textile is immersed in a bath containing an antimicrobial dye or molecule, and usually heated so the active component diffuses into or chemically bonds with the fiber. In practice this may involve adding electrolytes or alkali (for reactive compounds) and raising temperature, much like reactive dyeing.^74^ Dip coating is a widely adopted technique for depositing thin, uniform layers onto various substrates, including textile materials. In this approach, the fabric or target surface is immersed in a solution that contains the functional coating agent, such as metal nanoparticles, intended to impart antibacterial activity and then withdrawn at a regulated rate. The withdrawal speed plays a critical role in determining the thickness and uniformity of the deposited film.^75^ The exhaust dyeing technique employed to functionalize the cotton fabric with the reactive antibiotic dye. To prepare the dye bath, 6.5 g of the reactive antibiotic (or 3.25 g each when two antibiotics were combined) was dissolved in 1.2 L of deionized water. The solution was then supplemented with 0.5 mL of Triton X-100 (octylphenol ethoxylate; Dow Chemical Co., Midland, MI) to act as a wetting and dispersing agent, 75 g of sodium sulfate to serve as the electrolyte, and 12 g of sodium hydroxide would be dissolved in 100 mL of concentrated water to promote effective dye fixation. The samples of cotton fabric, each weighing 20 g were submerged in the dye solution and kept for 30 minutes with the temperature of 60 °C to facilitate even adsorption of the dye. The fabric was then subjected to elevated temperature of 80 °C for at least 30 min to ensure complete dye exhaustion and covalent bonding of the reactive antibiotic to the cellulose hydroxyl groups. After dyeing, the fabrics were washed several times with deionized water, maintaining the temperature at 80 °C with the time duration of 10 min to eliminate any unbound dye, and finally dried in an oven at 105 °C. For example, reactive antibiotics were covalently attached to cotton *via* an exhaust process. The cotton was incubated in a warm solution of the reactive antibacterial compound, then alkali and heat were applied to bind the agent to the fabric.^76^ In a recent study, a cotton reactive dye was synthesized by linking an antibacterial moiety to a dye molecule; this was applied by standard exhaust dyeing and produced 99.99% bacterial kill against *E. coli* and *S. aureus*, retaining full activity even after 20 industrial laundering cycles. In general, exhaust dyeing allows high uptake and fixation of functional molecules without the need for rollers, but may result in deeper penetration into fibers. The technique ensures better penetration but consumes more chemicals and water.^77^

### 4.3 Sol gel coating

In the sol gel prepartation and its coating over the fabric structure, the coated material starts with forming a liquid “sol” by combining metal alkoxide precursors (such as silanes) with water, or organic solvent alcohol, and sometimes acid or base catalysts. In this stage, hydrolysis converts alkoxy groups with hydroxyl groups, after which condensation reactions generates M–O–M (*e.g.*, Si–O–Si) network, which results to gradually increase viscosity of solution. This partially semi-condensed sol is subsequently applied to fabrics through dip-coating, padding, or spraying to achieve a uniform deposition on textile surfaces.^64^ After coating over textile, the excessive amount of liquid is removed by squeezing in padder and the fabric is dried to evaporate solvents and then allow further condensation. Sometime a curing step also involves, which normally converts the deposited sol into a thin oxide or hybrid network that bonds firmly to the textile. The important parameters such as precursor type, catalyst concentration, water to alkoxide ratio, solvent system, viscosity, reaction time, and curing temperature critically determine coating uniformity, adhesion, porosity, and mechanical flexibility effects the process and the finished material.^78^ In a recent research work, cotton fabrics were coated with sol gel process (utilized a low-temperature sol–gel) by TiO_2_ particles to achieve the antimicrobial effect. In their procedure titanium(iv) tetraisopropoxide was hydrolyzed in acidified aqueous medium (water medium) at roughly 80 °C for eight hours to form a stable TiO_2_ hydrosol. The cotton textiles were first underwent the scouring (sodium carbonate treatment) to remove surface impurities and then dried. Also a strong crosslinker comprising 1,2,3,4-butanetetracarboxylic acid and sodium hypophosphite was applied at 70 °C for two hours to promote covalent bonding between the cellulose matrix and TiO_2_*via* ester linkages. Following treatment, the fabrics were washed, dried at 60 °C, and evaluated for coating durability through standardized washing protocols.^79^ The TiO_2_-coated cotton fabrics showed strong antibacterial effectiveness due to the photocatalytic action of the sol–gel-deposited nanoparticles. Once activated by light, the TiO_2_ generated reactive species that damaged bacterial cells and prevented their growth. Overall, the sol gel treatment produced a durable antibacterial textile with sustained activity and good surface coverage of TiO_2_, making it an efficient antimicrobial fabric system (Fig. 1(c)).^79^

### 4.4 Solution spinning

Solution spinning is an effective technique for developing antibacterial fabrics because it allows antimicrobial agents to be incorporated directly into the polymer solution before fiber formation, ensuring uniform distribution and strong entrapment within the fiber matrix. In this process, the polymer is dissolved in a suitable solvent, mixed with antibacterial agents and then extruded through a spinneret into a coagulation bath (wet spinning) or evaporative chamber (dry spinning) to form solid fibers.^80^ Wet spinning of cellulose acetate (CA) solution embedded with AgNPs was done.^81^ The thickness of fiber (270 *vs.* 300 µm) and mechanical integrity remained unchanged. For the preparation of CA@AgNPs solution, the spinning solution was loaded into a 12 mL disposable plastic syringe (NORMJECT®, Henke Sass Wolf, Germany) and mounted on a syringe pump. With the help of syringe pump (Fusion 710, Chemyx Inc., USA), the solution was extruded at a constant flow rate of 1 mL h^−1^. A syringe, plastic tubing, and a 23-gauge needle (inner diameter ≤330 µm) were connected to the setup, allowing the solution to exit through the needle and be extruded directly into a 0.1 M NaOH coagulation bath. After an appropriate deacetylation time duration, the resulting fibers were collected, thoroughly rinsed with deionized water, and air-dried at ambient conditions.^81^ In another experimental work, composite of cellulose, was treated with silver nanoparticles (AgNPs) and alkaline lignin (AL) was fabricated using a wet-spinning technique. The preparation began by dissolving AL and cotton cellulose (CC) in an *N*,*N*-dimethylacetamide/lithium chloride (DMAc/LiCl) system, followed by wet spun of the mixture to form AL/CC fibers. The AL/CC dope was extruded through a spinneret at a feed rate of 0.4 mL min^−1^ into a deionized water coagulation bath, which maintained at room conditions (23 °C, 50% RH). AgNPs were then produced *in situ* on the surface of the AL/CC-g fibers, producing Ag/AL/CC-g composite fibers. These fibers demonstrated excellent antibacterial performance, achieving more than a 99.99% reduction in *E. coli* and *S. aureus* populations^82^ (Fig. 1(d)).

### 4.5 *In situ* nanoparticle synthesis

**Fig. 2:**
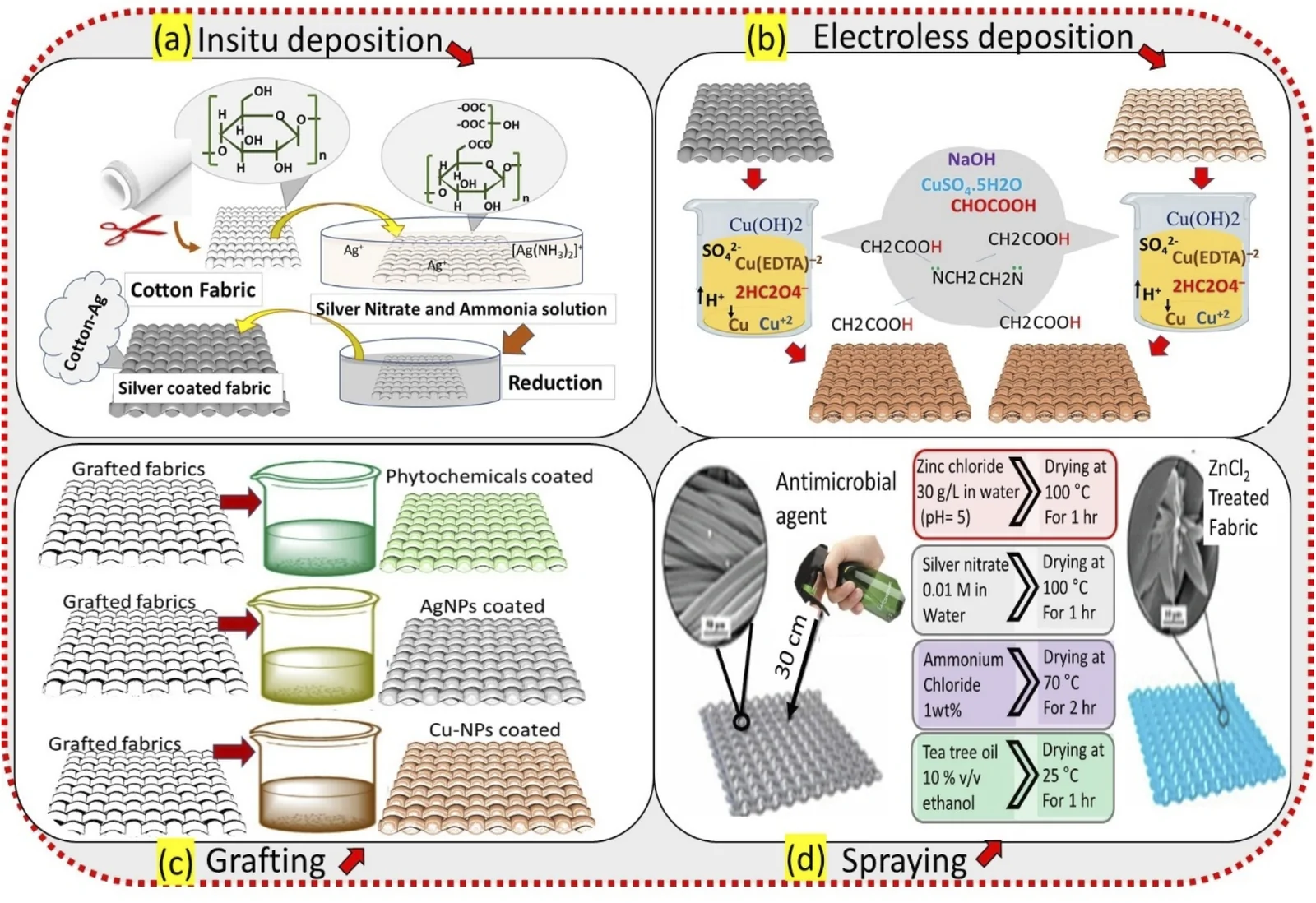
(a) *In situ* deposition of silver over the fabric structure. Redrawn with permission from [Ali], [silver-plated stretchable elastomeric electrodes for electrotherapy applications]; published by [industrial textile], [2024].^88^ (b) Copper electroless plating over silver and copper particles coated fabrics. Redrawn with permission from [Azam], [a comparative performance of antibacterial effectiveness of copper and silver coated textiles]; published by [industrial textile], [2022].^92^ (c) Deposition of copper, silver and copper over the grafted fabric. Redrawn with permission from [Farrukh], [the comparative performance of hytochemicals, green synthesised silver nanoparticles, and green synthesised copper nanoparticles-loaded textiles to avoid nosocomial infections]; published by [nanomaterials], [2022].^12^ (d) Spraying of antimicrobial agent on fabric. Redrawn with permission from [Vojnits], [advancing antimicrobial textiles: a comprehensive study on combating ESKAPE pathogens and ensuring user safety]; published by [MDPI], [2024].^96^

*In situ* nanoparticle synthesis refers to either direct production of nanoparticles or within the textile substrate by introducing a precursor (*e.g.*, metal salts) followed by its reduction, hydrolysis, or thermal conversion on the fabric matrix, rather than synthesizing nanoparticles separately and then depositing them.^84^ Metal salts impregnated into fabric are chemically reduced to nanoparticles directly on the fiber surface. This approach produces strong interfacial bonding and improved wash durability.^84^ In a recent research work, a simple *in situ* strategy was used to create antimicrobial textiles by forming silver nanoparticles directly on fiber surfaces rather than depositing pre-made particles. Fabrics are first soaked in a silver nitrate solution so Ag^+^ ions diffuse into or adsorb onto the fibers, then transferred to a sodium borohydride bath where the ions are reduced in place, producing metallic nanoparticles that grow and anchor tightly within the textile structure. Because the nanoparticles form on-site, they show stronger adhesion, less leaching, and more uniform distribution compared to *ex situ* coating methods. This method demonstrates that these *in situ* synthesized Ag-NPs impart strong antibacterial activity against *Bacillus subtilis* with natural fibers like wool and raw cotton showing the highest nanoparticle loading and most durable antimicrobial effect. Even after oxidative stress or washing, the *in situ* generated nanoparticles remain attached sufficiently to maintain bactericidal performance, whereas synthetic fibers, with fewer binding sites, show lower retention but still exhibit measurable antimicrobial activity.^85^ Azam Ali *et al.* developed very simple and low-cost route for *in situ* deposition of copper and silver nanoparticles over the cotton and polyester fabrics.^62,86^ In another work, they simply dipped the textiles into the salt solutions then reduced it in strong reducing agent (sodium dithionate) solution. The deposition was homogenous and resultant fabrics were effective against both kind of bacteria (Gram positive and Gram-negative bacteria).^87^ Azam Ali *et al.* recently deposited the silver nano-particles by *in situ* deposition method over the cotton substrate. At first fabric was impregnated into silver salt solution then carried out reduction with mild reducing agent. They claimed a clear zone of inhibition around the textile surface during antibacterial qualitative analysis. Fig. 2(a) is showing the schematics for insitu deposition of silver over the fabric structure while.^88^

### 4.6 Electroless plating of textiles with antibacterial metal and ions

Electroless plating, also called as an autocatalytic plating, is chemical reduction process that deposits a metallic coating on a substrate *via* autocatalytic reactions, independent of external electrical power. Instead, using a reducing agent in solution which could generate metal ions into a solid metallic layer, it directly works on the surface of the material (*e.g.*, text, wiles, polymers, metals, ceramics).^89^ This process is mostly used for silver coatings on synthetic fibers, providing antimicrobial conductivity. The process requires precise control of pH and catalyst deposition.^90^ Electroless plating has emerged as a reliable technique for imparting antibacterial functionality to textiles through the uniform deposition of metallic layers without the need for an external power source. In this process, metals such as copper (Cu), silver (Ag), and zinc (Zn) are chemically reduced and deposited onto fabric surfaces. These metals are well known for their antimicrobial activity: silver interferes with bacterial cell membranes and metabolic pathways, copper exhibits both antibacterial and antiviral effects, and zinc contributes to sustained antimicrobial performance while improving coating stability. The procedure typically begins with surface pre-treatment to enhance fabric reactivity and promote strong metal adhesion. The textile is then immersed in a plating bath containing metal ions and a suitable reducing agent, enabling controlled metal deposition. Beyond conferring antimicrobial properties, electroless plating can improve mechanical robustness, laundering durability, and overall wear resistance. Subsequent advancements in this field have focused on *in situ* growth of Ag and Cu nanoparticles within the textile matrix itself. By occupying the inter-fiber spaces and forming interconnected conductive pathways.^87^ Ali *et al.*^91^ further examined the surface metallization of cotton using Ag and Cu nanoparticles through an electroless approach. The fabrics were first activated to create nucleation sites by depositing initial silver and copper particles, followed by controlled plating to form a thin copper layer (Fig. 2(b)).^92^

### 4.7 Grafting modifications

The grafting method involves the attachment of functional polymers to fabric surface by covalent bonding such as hydrophobicity, antimicrobial activity, UV resistance, drug delivery capabilities, flame resistance, and improved dyeability. A range of grafting techniques, including chemical, plasma-induced, and radiation-induced grafting, have been explored to achieve these modifications. Different grafting techniques such as radiation-based processes, including chemical, plasma-induced, have been explored to introduce these enhancements. In a recent study, a two-step modification strategy (oxidation followed by grafting quaternary ammonium salts) was developed to functionalize cotton fibers for durable antibacterial performance. The modified cotton showed >99% antibacterial efficiency against *E. coli*, *S. aureus*, and *C. albicans*, with strong retention of activity even after 100 washing cycles. Blended into nonwoven fabrics, even 10% modified fibers provided high antibacterial activity while maintaining biocompatibility, spinnability, and low-cost scalable processing for healthcare textiles.^93^ In fact, in most of the grafting process, the reactive functional groups are generated over the fabric structure which inturn make bond with antibacterial agents (covalently). The benefits of this technique as it is free of solvent or solutions but requires requires costly equipment.^94^Fig. 2(c) represent the different coating techniques for textiles.

### 4.8 Spraying the antibacterial agents over the textile structure

The spraying of antibacterial agents over the fabric surface provides an efficient and scalable functionalization of textiles also efficient deposition method, without compromising the softness, tactile qualities and air permeability of the textiles. The process involves, uniformly spray of liquid mixtures composed of nanoparticles, biocides, natural extracts or polymer-based antimicrobial agents over the fabric surface. It ensures controlled surface coverage and deep penetration into the fabric structure.^95^ Studies have shown that spray deposition of silver nanoparticles provide a uniform deposition and same antibacterial activity (on each part of fabric), while fine spray conditions parameters significantly improve nanoparticle attachment and long-term functional stability. Recent progress further emphasizes the effectiveness of both natural and synthetic antimicrobial agents and ensure their durability when applied through spray coatings. Overall, spraying represents a versatile and industry-friendly route for developing next-generation antibacterial textiles suitable for healthcare, protective wear, and smart wearable applications.^14^ The composite of the pigment-binder and antibacterial agent (Ag nanoparticles) was considered an easy-to-manage approach for creating coated fabric with antibacterial properties by spray coating process. The coating was very smooth and less agglomerated.^95^

The method of spraying enables allows antimicrobial agents to be deposited in a controlled and even manner, on intricate textile geometries or three-dimensional fabric surfaces. A recent research study used spray coating on the circular textile swatches with the diameter 4 cm. The fabric materials were immersed in a bath containing 1 wt% of a commercial detergent for 30 min, then rinsed twice at 50 °C to eliminate residual impurities.^5^ A range of antimicrobial formulations such as metal agents, quaternary ammonium compounds (QACs), and alternatives derived from plants, were subjected to washed fabric swatches by scalable spray coating method. Zinc chloride (ZnCl_2_) spray was prepared with ZnCl_2_ in 30 g L^−1^ of water and lowering the pH to 5 with acetic vinegar, whereas a 0.01 M AgNO_3_ solution served as the silver source.^5^ The quaternary ammonium compounds (QAC) such as trimethoxysilylpropyl, octadecyldimethylammonium chloride (HM4072) and trihydroxysilylpropyldimethyl octadecyl ammonium chloride (HM4005) were diluted to 1 wt% in methanol and water, following the manufacturer's guidelines. Tea tree oil was diluted in 10% v/v of ethanol prior to application. Each antimicrobial solution was applied at a uniform rate of 0.5 L m^−2^ using a power hand sprayer. To ensure complete and even coverage, the spray and drying cycle was repeated twice, with a maintaining distance of 30 cm between the nozzle and the fabric surface, as illustrated in Fig. 2(d)

### 4.9 Comparative analysis of curing technologies for antibacterial textile fabrication

Various curing and fixation technologies have been investigated to enhance the adhesion, durability, and long-term antibacterial performance of functional textile coatings. Conventional thermal curing through the pad-dry-cure method remains the most widely adopted industrial approach because of its operational simplicity, scalability, and ability to provide strong fixation of antibacterial agents *via* crosslinking reactions. However, the process is associated with high energy consumption, significant water and chemical usage, and the potential thermal degradation of sensitive bioactive compounds.^97^ In recent years, alternative curing strategies have attracted increasing attention due to their improved energy efficiency and compatibility with advanced wearable textiles. UV curing enables rapid photopolymerization at relatively low temperatures, thereby preserving the flexibility and breathability of heat-sensitive fabrics while reducing processing time and energy demand.^98^ Plasma-assisted curing and surface activation methods further improve coating adhesion and wash durability by generating reactive surface species without extensive solvent or water consumption, making them highly attractive for environmentally sustainable smart textile manufacturing.^99^ Similarly, microwave-assisted curing offers rapid volumetric heating with lower thermal stress on textile substrates, whereas infrared curing provides faster and more energy-efficient surface heating compared with conventional convection systems.^15^ Electron beam curing has also emerged as an advanced solvent-free technology capable of producing excellent coating fixation and wash stability without the need for thermal initiators, although its industrial adoption remains limited by high equipment cost and radiation safety concerns.^100^ Collectively, these curing technologies exhibit different balances between processing efficiency, environmental impact, antibacterial durability, and suitability for wearable textile applications, as summarized in Table 3.

**Table 3:** Comparative analysis of curing methods used in antibacterial textiles for wearable applications

Sr. no.	Curing method	Principle	Advantages	Disadvantages	Durability	Wearable textile suitability	Ref.
1	Conventional	Pad-dry cure heat fixation	- Industrially established	- High energy use	High wash durability	Suitable for cotton/blends	97
	Thermal curing		- Strong fixation	- Possible biomolecule degradation			
			- Bulk production	- High water/chemical consumption			
2	UV curing	UV-induced grafting	- Rapid processing	- Limited penetration	Good durability	Highly suitable; maintains flexibility	98
			- Low-temperature treatment	- Requires photoinitiators UV safety concerns			
			- Energy efficient				
3	Plasma-assisted curing	Plasma activation for coating adhesion	- Eco-friendly	- Expensive equipment	Excellent wash durability	Highly suitable for smart textiles	99
			- Minimal water use	- Optimization required			
			- Enhanced adhesion				
4	Microwave-assisted curing	Microwave-induced volumetric heating	- Fast curing	- Non-uniform heating	Moderate–high durability	Suitable for sensitive wearable fabrics	15
			- Energy efficient	- Specialized equipment needed			
			- Reduced thermal stress				
5	Electron beam (EB) curing	Electron beam induces polymerizatio	- Solvent-free	- High equipment cost	Excellent durability	Suitable for advanced smart textiles	100
			- Rapid curing	- Radiation safety concerns			
			- Excellent adhesion				
6	Infrared (IR) curing	IR radiation provides rapid surface heating	- Faster drying	- Limited penetration	Moderate durability	Suitable for lightweight fabrics	100
			- Energy saving	- Overheating risk			
			- Controlled heating				

## 5 Smart antibacterial fabrics

To improve durability and reduce environmental risks, recent developments aim to enhance bonding between antibacterial agents and fibers, use microencapsulation techniques, or incorporate hybrid inorganic–organic systems. Nonetheless, the shift is gradually moving toward sustainable and bio-based antibacterial textiles, using natural extracts, biopolymers, and green synthesis routes to replace conventional approaches. Smart fabrics and textiles and are those that are prepared to detect environmental changes and could adapt or respond to those external stimuli. (for example thermal, mechanical, chemical, magnetic cues).^8^ Smart antibacterial fabrics are advanced textile materials designed not only to inhibit or eliminate microbial growth but also to sense, adapt, and respond to environmental or biological stimuli. These fabrics can regulate the release of antimicrobial agents, signal changes in antibacterial performance, or activate antimicrobial action in response to the presence of pathogens.^101^ A category of smart fabric was prepared by the integration of drug carrier which are stimulus responsive, thereby having significance for personalized therapeutic applications. These materials can function as both topical and transdermal drug delivery platforms. They have been gaining great importance in aromatherapy, antimicrobial treatments, pain management, hormone therapy, and the management of skin conditions such as acne, psoriasis, atopic dermatitis, and melanoma.^102^ Hence, smart textiles serve as a key functional component in systems designed for transdermal therapy and advanced wound care.^103^

### 5.1 Mechanisms of action

Smart antibacterial textiles mark a transition from traditional, customary antimicrobial methods to systems that are capable of self-regulating or stimuli responsive behavior. Instead of constantly releasing biocides, these materials are designed to sense environmental or biological cues such as changes in temperature, pH, moisture, or bacterial presence and also activate their antimicrobial function only when needed. Their performance relies on the integration of functional materials (such as nanoparticles, responsive polymers, or photosensitive compounds) that interact with these cues to control or enhance antibacterial activity in a targeted, efficient manner.^5^

#### 5.1.1 Stimuli triggered release

Microcapsules (having drugs) or hydrogel matrices (loaded with antimicrobial agents); respond to change in pH, temperature, or moisture by swelling or by rupture or linked to bacterial metabolism or perspiration. For example, an encapsulated silver salts with in a pH triggered polymer shell can emit Ag^+^ ions under acidic microenvironment formed by bacterial colonies.^104^

#### 5.1.2 1Photocatalytic and photothermal activation

Under light irradiation the activated substances such as TiO_2_, ZnO, or carbon-based nanostructures can generate reactive oxygen species (ROS), that effectively kill bacteria killing bacteria while simultaneously degrading organic contaminants. Doped TiO_2_ with transition metals such as Mn, Fe, V, Cu is a visible light active substance can generates localized electronic states, which in turn narrow the bandgap and shift the absorption band into the visible range. Moreover, if coupling TiO_2_ with plasmonic metals like Ag nanoparticles activates surface plasmon resonance under visible light, facilitating hot electrons to tranfer into the conduction band of TiO_2_ and hence enhancing ROS formation. Such doped or hybrid photocatalysts provide photocatalytic response aligning well with visible light ranges relevant to indoor wear.^105^

#### 5.1.3 Electrical stimulation and contact killing

Electrically conductive textiles embedded with carbon nanotubes, graphene, or metal nanowires can generate localized electrical fields that are capable of damaging bacterial membranes when low voltage is applied. This electrodynamic mechanism enables simultaneous sensing and active sterilization. Actually, carbon nanosheets possess sharp edges that mechanically cut or damage bacterial membranes acting as “nanoknives” or “nanoblades” and causing permanent leakage of internal components. Simultaneously, electron transfer from the conductive material to the bacterial membranes, disturbing the redox balance and promotes the formation of the reactive oxygen species (ROS). This oxidative stress further damages cellular proteins and lipids, leading to bacterial inactivation.^106^

#### 5.1.4 Sensor integrated feedback

Hybrid smart fabrics embed biosensors that detect microbial load or physiological changes and trigger control release *via* microheaters or voltage pulses. For example, a micro heater or voltage driven surface is activated to release antimicrobial agents or apply an electrical, thermal stimulus that disrupts bacterial adhesion, biofilm formation or viability. Such closed looped: sensor actuator platforms extend beyond passive antimicrobial coatings by combining real time monitoring with active intervention offering higher specificity, reduced agent use, and the potential to avoid antimicrobial resistance. Such self-regulating systems form the foundation of responsive wearable therapeutics smart textiles.^107^

### 5.2 Agents used in smart antibacterial textiles

The functional agents in smart textiles are typically multifunctional composites rather than single chemicals. Selection of agents also mostly based on the fabrication technique. The Table 4 is showing the examples of smart antibacterial agents and their activation modes.

**Table 4:** Examples of smart antibacterial agents and activation modes

Agent type/system	Mechanism	Trigger or control parameter	Advantages	Ref.
Polyacrylonitrile (PAN) having triclosan	- Electrospun nanofiber	- Moisture exposure	- Antibacterial and fire-retardant	108
	- Enables sustained antibacterial activity	- Bacterial contact	- Improved moisture transport	
	- Enhanced hygroscopicity			
Gardenia yellow natural dye inkjet printed	- Antibacterial natural dye	- pH, surface tension	- Eco-friendly, stable	109
	- Dye entrap in polyol solvent-modified ink and sodium polyacrylate fixation	- Viscosity	- Non-polluting inkjet process	
Ag@polymer microcapsules	- Release of Ag^+^ to destroy (bacterial metabolism)	- pH responsive	- Stimuli-responsive release enabled by the polymeric carrier system	110
			- Reduced leaching	
PNCS (poly(*N*-isopropylacrylamide)/chitosan) hydrogel	- Controlled Ag^+^ release from PNCS matrix	- Temperature and pH responsive	- Dual responsive (temperature & pH)	111
Cyclodextrins, aza-crown ethers	- Host–guest inclusion	- Diffusion rate	- Sustained release	112
	- Controlled drug delivery	- Concentration gradient	- Suitable for cure dermatitis	
Neicosane core and CuO-doped polyurea shell	- Phase-change induced heat absorption/release combined with CuO-based	- Temperature variation	- Thermoregulation with antibacterial	113
			- Superhydrophobic	
CaF_2_-NPs @ acrylate copolymer paste	- Contact-based antibacterial activity through gradual ion release	- Responsive to bacterial contact	- Excellent durability; >93% bacterial reduction	114
Quaternary ammonium salt/MXene nanosheet	- Electrostatic self-assembly and photothermal antibacterial effect	- Light exposure	- Smart, wearable, and conductive cotton fabric	115
		- Temperature responsive		
Polysiloxane	- Photodynamic and contact-killing coating	- Light exposure	- >98% bacterial reduction	115
		- Responsive		
poly(*N*-isopropylacrylamide)@Si-QAC	- Bio-barrier *via* quaternary ammonium immobilized in PNCS microgel carrier	- Concentration-dependent effect	- Long-lasting bio-barrier protection on cotton fabric	116
TiO_2-_Fe_3_C-Fe-Fe_3_O_4_/graphitic carbon	- Photocatalytic	- Visible light irradiation	- Dual function: Adsorption + photocatalysis	117
	- Graphitic carbon supports adsorption (π–π)			

### 5.3 Fabrication techniques of smart antibacterial fabrics

Smart antibacterial textiles demand fabrication routes that ensure both durability and functionality integration. The major approaches include.

#### 5.3.1 Electrospinning

**Fig. 3:**
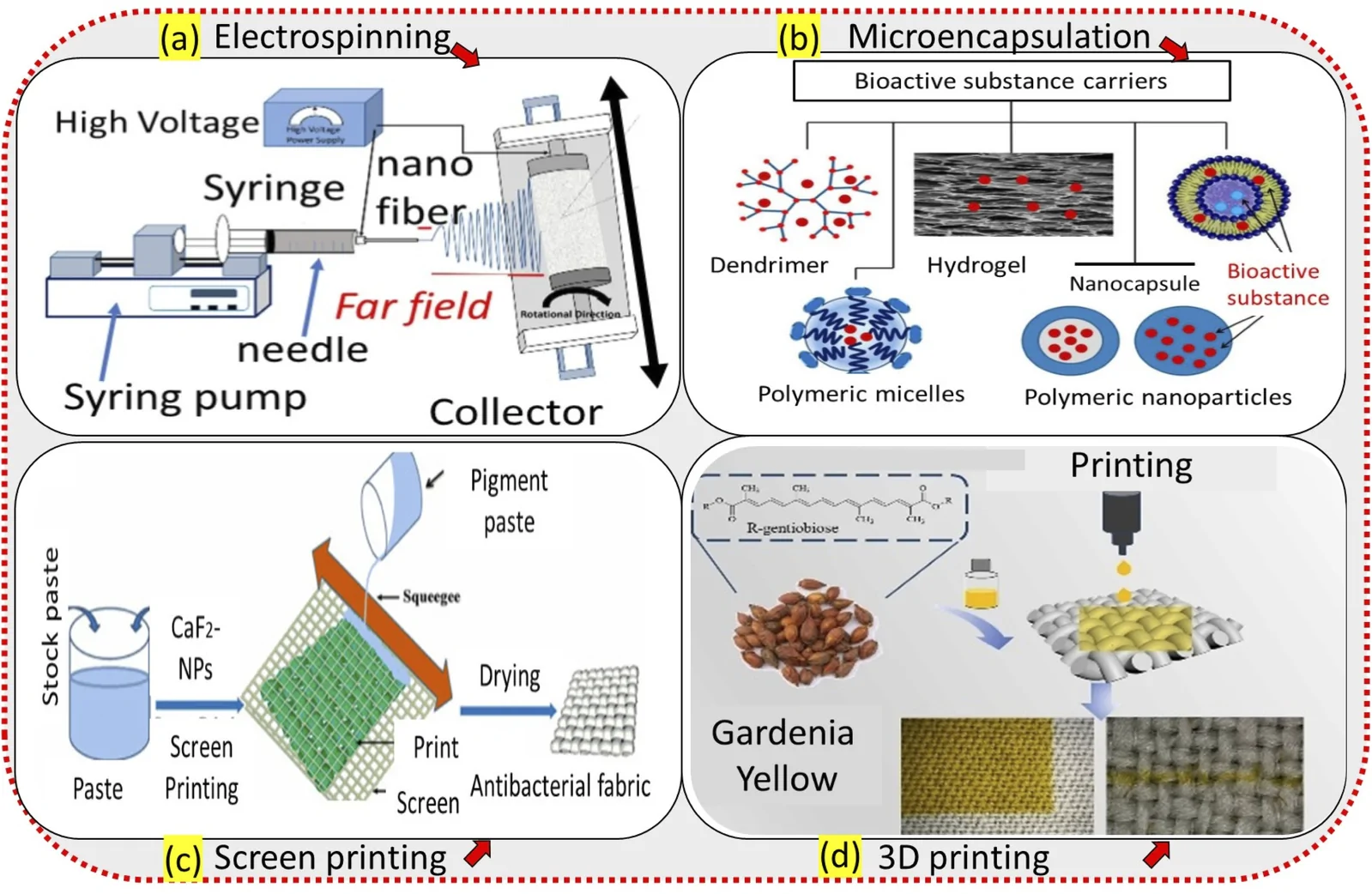
(a) Schematic representation of a solution electrospinning setup. Redrawn with permission from [Bonakdar], [highly porous biobased membranes *via* electrospinning of PBS and CTAB]; published by [Elsevier], [2023],.^126^ (b) Encapsulated antibacterial agent a suitable carrier and controlled release. Redrawn with permission from [Atanasova], [textile materials modified with stimuli-responsive drug carrier for skin topical and transdermal delivery]; published by [MDPI], [2021].^103^ (c) Coating of CaF_2_-NPs on textile. Redrawn with permission from [Kumar], [screen printed calcium fluoride nanoparticles embedded antibacterial cotton fabric]; published by [Elsevier], [2022],.^114^ (d) Gardenia yellow ink inkjet printing process.^109^ Redrawn with permission from [Wang], [Green fabrication of inkjet printed antibacterial wool fabric with natural gardenia yellow dye]; published by [Elsevier], [2023].^109^

It is a versatile and widely used nanofabrication method to produce ultrafine fibers (typically in the nanometer to micrometer range) from polymer solutions or melts under a strong electric field. The process involves ejecting a charged polymer jet from a needle toward a grounded collector, forming continuous fibers with high surface area, porosity, and tunable morphology. These features make electro spun nanofibers excellent candidates for biomedical, filtration, and antibacterial applications.^118^ Schematic representation of a solution electrospinning setup is shown in Fig. 3(a). In fact; nanofiber membranes containing embedded nanoparticles or drugs exhibit high surface area and controllable porosity. Multi-layer electrospinning enables gradient release profiles or co-axial core–shell structures. For example, electrospun polycaprolactone loaded with AgNPs maintained >95% antibacterial efficiency after 50 wash cycles. However, electrospinning remains limited to small-scale production.^119^ As electrospinning technique allows for precise integration of functional nanofibers within the fiber matrix; which are difficulties with conventional spinning or coating methods. Electrospinning produces ultrafine nanofibers with the highest surface area and interconnected porosity, which enhance both moisture transport (hygroscopicity) and interaction with antibacterial agents.^108^ Therefore, a study was aimed on the development of a novel fire-retardant textile that possess antibacterial functionality as well as high moisture absorption characteristics the textile was manufactured with a blend of Kevlar® para-aramid fibers, providing inherent flame resistance, and flame-retardant viscose. Subsequently, polyacrylonitrile (PAN) having triclosan (antibacterial agent) were coated over Kevlar® para-aramid fibers by electrospinning deposition.^108^ Veeramuthu *et al.* developed bio-inspired electrospun quantum nanostructured textiles exhibiting enhanced photothermal antibacterial performance, filtration efficiency, and self-powered theranostic functionality for intelligent wearable applications. The study demonstrated that the incorporation of sustainable quantum nanostructures within electrospun fibrous matrices significantly improved bacterial inhibition while enabling high-quality factor sensing for respiratory monitoring and healthcare diagnostics.^120^

#### 5.3.2 Microencapsulation and layer-by-layer assembly on textiles

Antimicrobial agents encapsulated within polymeric shells or polyelectrolyte multilayers can release under environmental triggers. The approach improves reproducibility and wash durability compared with direct coating. Capsule rupture may still lead to uneven distribution with the passage of time (Fig. 3(b)).^103^ Microcapsules incorporated onto fabrics are widely used to enable the sustained release of antibacterial agents. A study conducted by Hans Christian Korting *et al.* highlights the use of textiles functionalized with cyclodextrins, fullerenes, aza-crown ethers ion-exchange fibers, hollow fibers, and various nanoparticles as drug delivery platforms for managing conditions such as atopic dermatitis, acne, psoriasis, and melanoma.^112^ Massella *et al.* expand the horizon for diverse drug carriers which includes nanospheres, micro and nanocapsules, inorganic nanoparticles, liposomes, and micro-hydrogels into textile systems for therapeutic applications.^121^ Another, research work was carried out to fabricate the phase-change material (PCM) based on smart textiles that possess both thermal energy storage capability and antimicrobial functionality.^5^ By using a one-step interfacial polymerization approach, researchers developed microcapsules composed of a *n*-eicosane core and a CuO-doped polyurea shell, exhibiting a hierarchical, dendritic morphology. These microcapsules demonstrated excellent thermal stability during energy storage processes.^113^ The superhydrophobic nature of these materials evidenced by a contact angle greater than 148°, which further enhances their suitability for textile integration, and provide improved thermoregulation and increased durability.^103^ Biofunctional textile system also align with advancement in drug-delivery technologies and also in the development of innovative strategies for transdermal administration of bioactive compound.^122^ There are some negative effects, if active agents are directly attached to the fabric surface and can also compromise their biological functionality, limit controllability, and create challenges in skin compatibility. Unlike conventional antibacterial textiles fabricated through surface deposition of synthetic antimicrobial agents, the methodology proposed by Kunnas *et al.* integrates antimicrobial functionality directly into renewable bio-based textile matrices, resulting in improved sustainability, structural compatibility, and durable antibacterial performance.^123^ Consequently, current approaches favour encapsulating biologically active substances (BAS) within carriers that fulfill specific requirements for regulated release and targeted activity.^124^

#### 5.3.3 Printing and 3D deposition

Conductive or photocatalytic inks containing nanoparticles are printed on textile substrates to form patterned antibacterial zones. Inkjet or screen printing allows integration with sensing circuits but requires precise rheology control.^5^ Screen printing is another widely used method for producing antimicrobial textiles. In this technique, a patterned stencil is prepared on a fine mesh screen, allowing selective deposition of antimicrobial inks or pastes onto the fabric surface. This process allows antimicrobial formulation to pass through the open areas of the mesh to create precise, uniform, and repeatable coating patterns across the textile. The antimicrobial ink, containing active ingredients like nano-Ag or other antimicrobial compounds, is then pushed through the screen onto the fabric using a squeegee. This results in the deposition of the antimicrobial agent in a controlled and patterned manner, according to the design of the stencil.^5^ After printing, the fabric undergoes a drying and curing process to ensure the antimicrobial properties are fixed and durable. Screen printing allows for the application of antimicrobial coatings in specific areas or patterns, making it ideal for textiles requiring localized protection or decorative antimicrobial features. A recent study explored the incorporation of calcium fluoride (CaF_2_) nanoparticles into a stock paste formed by crosslinkable acrylate copolymer.^114^ The formed material was then applied to cotton fabric using a screen printing technique, which employed a 165-mesh screen to ensure uniform distribution of the paste. After coating, the CaF_2_ treated cotton (Cot-CaF_2_) was dried and then cured at 120 °C for 5 and 7 min, respectively. The screen coating process is illustrated in Fig. 3(c). Moreover, the cotton samples were prepared using only the stock paste, to examine the efficacy of the binder and other formulation components. The produced nanoparticle treated fabric exhibited impressive wash durability, maintaining more than 93% bacterial reduction even after 15 repeated laundering cycles.^114^ Inkjet printing method represents one of the fastest and advance imaging technologies, which offers clear advantages over traditional printing methods, including reduced waste generation and lower consumption of energy and water.^125^ However, inkjet inks must be precisely prepared to achieve reliable droplet formation through fine printer nozzles at high operating speeds. Incorporating functional additives such as antimicrobial or fragrance releasing agents into an ink system could poses formulation challenges, such as pH, surface tension, viscosity parameter, and conductivity, which must remain within strict limits. The long-term stability and printability of digital inks are typically assessed by monitoring these properties over time^125^ In a study by Mengyue Wang, a water soluble, naturally derived gardenia yellow dye was used into an inkjet compatible ink and then printed onto wool fabrics.^109^ Several light scattering analysis confirmed that the dye ink possessed excellent stability (Fig. 3(d)). With the adjustment of the viscosity and surface tension with polyol-based organic solvents allowed the formation of stable droplets during printing. It is also speculated that sodium polyacrylate as sizing agents and polyol solvents influenced color depth and the sharpness of printed contours. Followed by a tannic acid treatment, the inkjet-printed wool fabric achieved satisfactory color fastness. In addition, the gardenia yellow dye imparted strong antibacterial properties, with reduction rates against *S. aureus* and *E. coli* exceeding 95%. This study explains that both the inkjet printing and color fixation steps used in this work are environmentally friendly, offering a promising route for creating natural, green, antibacterial textiles.^109^

#### 5.3.4 *In situ* growth of responsive nanostructures

The *in situ* growth approach refers to the direct formation of functional nanostructures on the surface of textile fibers, without the need for an intermediate coating or post-deposition step. This method allows the antibacterial nanomaterials to be firmly anchored onto the fabric surface, improving their durability, stability, and long-term functionality even after repeated washing or mechanical stress. Azam Ali *et al.* developed the antibacterial fabrics by insitu deposition of copper nanoparticles over the cotton fabric structure. The base fabric usually dip with several cycles into the salt solution, subsequently (the copper ions attached fabric) was dipped into the strong reducing solution.^62,91^ Infact a variety of the fabric substrate (such as cotton, polyester, or nylon) acts as a template or reactive platform, where nanostructures like metal nanoparticles (Ag, Cu, ZnO), metal–organic frameworks (MOFs), or stimuli-responsive nanolayers are synthesized directly through chemical reactions or reduction processes. These responsive nanostructures can exhibit stimuli-dependent antibacterial behavior respond to changes in pH, humidity, temperature, or light.^5^ A smart antibacterial textile with dual temperature and pH responsiveness was developed using a chitosan-based system.^111^ In this procedure, nanoparticles of silver metal were generated directly on the fabric surface through an insitu synthesis approach. The PNCS hydrogel which is composed of poly(*N*-isopropylacrylamide) and chitosan, could serve both as a functional coating and as a biosynthetic platform for producing nAg, enabling the creation of a responsive viscose fabric. Subsequently, two modification strategies were employed, one of them is the direct immobilization of nanoparticles onto PNCS-treated fibers and the second one is the *in situ* nanoparticle formation within the hydrogel-coated fibers. The *in situ* route resulted in a higher concentration of nAg, simultaneously improving UV protection and delivering strong antibacterial effects, with bacterial reductions exceeding 90% against *E. coli* and *S. aureus*. Acting as a reservoir, the nPNCS hydrogel also facilitated controlled nanoparticle release while maintaining moisture regulation.^111^

#### 5.3.5 Pad-dry or hybrid system for deposition of responsive agents

**Fig. 4:**
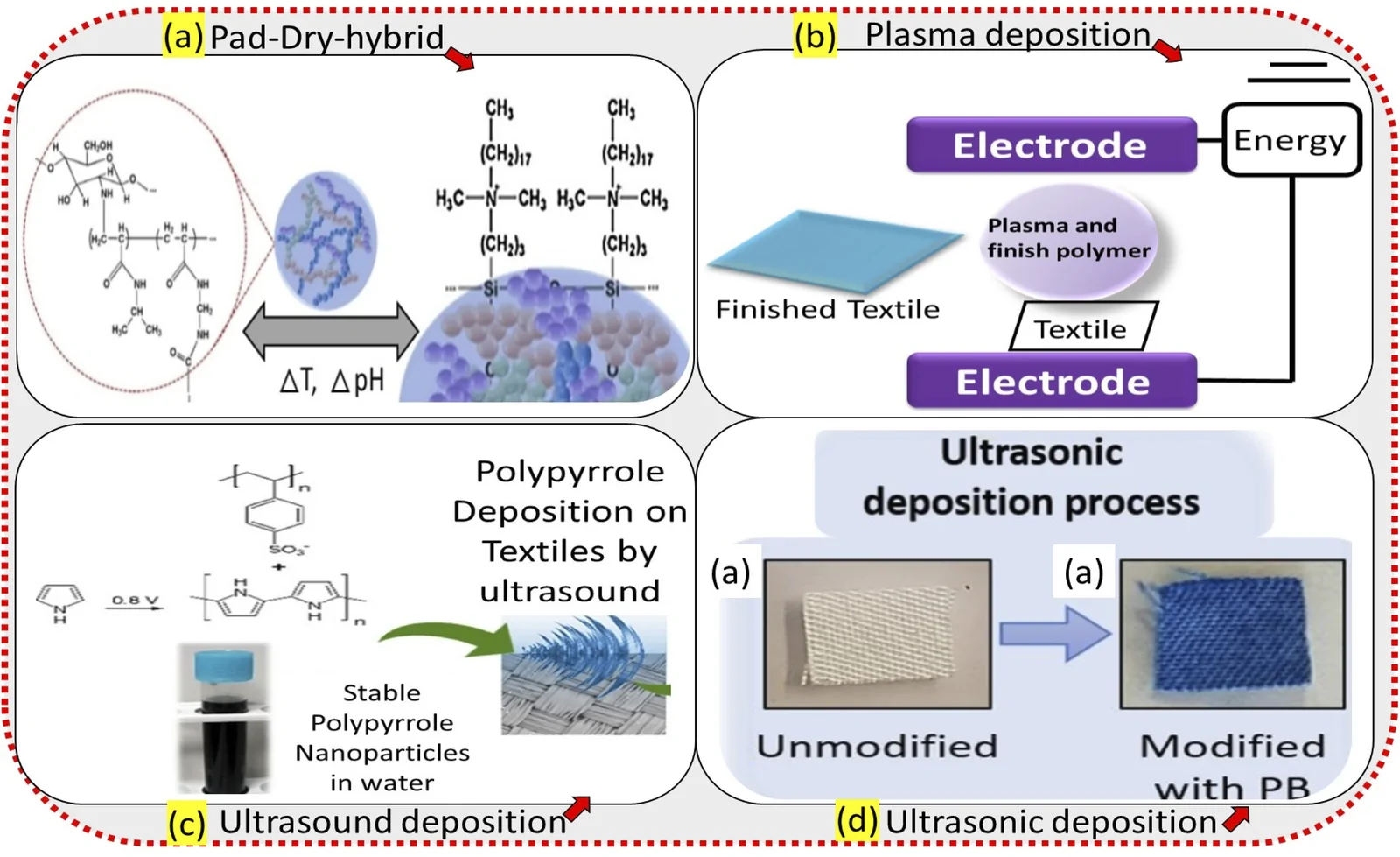
(a) Possible mechanism of PNCS hydrogel synthesis (PNCS stimuli-responsive active coating) by pad-dry-hybrid system on the cotton surface. Redrawn with permission from [Stular], [tailoring of temperature- and pH-responsive cotton fabric with antimicrobial activity: effect of the concentration of a bio-barrier-forming agent]; published by [Elsevier], [2021].^116^ (b) Plasma deposition of antimicrobial agents on textile surface. Redrawn with permission from [Cornelius], [atmospheric pressure plasma grafting of a vinyl-quaternary compound to nonwoven polypropylene and cotton]; published by [Sage], [2018],.^135^ (c) Ultrasound deposition of polypyrrole over the textile. Redrawn with permission from [D. O. Sanchez Ramirez], [antibacterial properties of polypyrrole-treated fabrics by ultrasound deposition]; published by [Elsevier], [2019],.^129^ (d) Polyester cotton fabrics unmodified and modified with Prussian Blue 0.25 mM by ultrasonic deposition. Redrawn with permission from [Vilanova], [smart sensing fabrics for live bacteria detection]; published by [MDPI], [2018].^133^

The pad-dry or hybrid system is a widely used method for depositing responsive antibacterial agents onto textile fabrics to impart durable and smart antimicrobial properties. In the conventional pad-dry process, fabrics are passed through a finishing bath containing antibacterial formulations such as silver nanoparticles, chitosan, or quaternary ammonium salts, then padded to control wet pickup, followed by drying and curing to fix the agents onto the fibers.^65^ Hybrid systems, on the other hand, integrate the pad-dry technique with advanced modification methods to improve durability, responsiveness, and release control of active agents. Such hybrid approaches may include pad-dry combined with sol–gel coating (for embedding metal nanoparticles in a silica network), pad-dry followed by plasma treatment (to enhance surface bonding and activation), or pad-dry coupled with layer-by-layer (LbL) self-assembly (to create multilayer responsive coatings). Other hybrid designs include pad-dry with graft polymerization or microencapsulation techniques, these, systems enhance the stability and reusability of antibacterial finishes while ensuring the fabric responds dynamically to environmental stimuli, making them ideal for advanced protective and healthcare textiles.^116^ The cotton fabric with the property of stimuli responsive characteristic was developed with the help of a dual-responsive microgel system composed of temperature and pH-sensitive poly-*N*-isopropylacrylamide (poly-NiPAAm) and chitosan (PNCS). This microgel acted as a carrier for the antimicrobial agent 3-(trimethoxysilyl)-propyldimethyloctadecyl ammonium chloride (Si-QAC), which forms a protective biofilm on the surface of fiber. With the help of pad dry cure technique, Si-QAC was employed onto 100% cotton fabrics in concentrations ranging from 0.05–4% to assess its antibacterial performance against *Staphylococcus aureus* (Gram-positive) and *Escherichia coli* (Gram-negative). Fig. 4(a) illustrate the most applicable procedure of PNCS hydrogel formation (PNCS and its function as a stimuli responsive active coating on cotton fibers using the pad dry hybrid method.^116^ In a related advancement, a recent study introduced a polysiloxane-based textile coating capable of two antimicrobial modes which encompasses passive contact inactivation mediated by amine/imine groups, and active photodynamic inactivation triggered by the generation of reactive oxygen species (ROS).^115^ With the help of padding process, the coating achieved over 98% inactivation of *E. coli* and methicillin-resistant *Staphylococcus aureus* (MRSA) within 24 h, with inactivation rate enhancements of 23-fold and 3-fold, respectively. These results demonstrate that dual-functional antimicrobial polymers can create durable, highly effective materials suitable for reducing pathogen transmission. Another key advantage of the padding process is that it helps maintain the fabric's comfort and resilience; two essential properties for textile sensors. Using this approach, smart wearable antimicrobial textile sensors for breath monitoring were developed.^127^ The novelty of this work lies in the development of wearable cotton-based fabrics capable of rapid photothermal antibacterial activity while simultaneously enabling real-time monitoring of subtle respiratory patterns. Using an electrostatic self-assemblage strategy combined with padding, chitosan based quaternary ammonium salts (HACC) were integrated with MXene nanosheets. The dense but flexible integration endowed the fabrics with excellent softness, excellent breathability, and stable electrical conductivity, enabling multifunctional antimicrobial performance in a comfortable wearable platform.

#### 5.3.6 Ultra sound coating

Ultrasound-assisted coating is very efficient technique for homogenize coating and preserves the mechanical softness and breathability of the fabric. Secondly, the technique offers a green, energy-efficient, and scalable approach to create durable, responsive antibacterial smart textiles suitable for medical, protective, and wearable applications.^128^ In this process, ultrasonic waves create acoustic cavitation within the coating bath, generating brief zones of high pressure and temperature. These micro-events enhance the dispersion, penetration, and bonding of antimicrobial agents onto fibers. This method accommodates the nanoparticles, biopolymers, and smart functional additives such as silver and zinc oxide particles, chitosan, or stimuli-responsive microgels and form uniformly deposition on fabrics without relying on aggressive chemicals or elevated processing temperatures.^129^ Copper-based nanoparticles (CuO, Cu_2_O) have also shown strong and long-lasting antibacterial performance. They can be incorporated into fabrics through both *in situ* and *ex situ* approaches, which include ultrasound assisted deposition and dip coating. These coatings typically retain their activity even after repeated laundering.^130–132^ A study conducted by D. O. Sanchez Ramirez *et al.* demonstrate that ultrasound assisted coating provides an efficient route for applying nanoparticles onto polyester fabric, producing a highly effective antibacterial finish it also reported that fabrics treated with approximately 4 g m^−2^ of polypyrrole achieved bacterial reduction values of 6.0 log for *Staphylococcus aureus* and 7.5 log for *Escherichia coli*.^129^ This approach is the combination of water based polypyrrole nanoparticle dispersions with continuous ultrasonic coating, which addresses several scale-up challenges associated with conventional *in situ* chemical deposition methods used for producing polypyrrole coated fabric material.^133^ This behavior is shown in (Fig. 4(c)).^129^ A smart textile for live bacteria detection of antimicrobial hospital tissues was proposed in another research. The capacity to detect viable bacteria was based on the use of Prussian Blue (PB) as electrochromic compound, with a clear reversible change of colour from PB to Prussian White (PW) after reduction from a bacterial metabolism process. PB nanoparticles were incorporated to polyester cotton fabrics by ultrasonic deposition. After performing different tests with bacterial samples of *E. coli* and *S.aureus*, a full colour change of the textiles was observed. These smart textiles will allow to determine the self-life of the antibacterial compounds as well to improve the control of hospital infection (Fig. 4(d).^133^ In another research, the fabrication of smart antibacterial textiles was achieved using a hybrid combination of the solution-dipping technique and ultrasound-assisted coating, representing a simple yet effective wet-chemical approach.^5^ In this process, ultrasonic energy was applied during the sequential dipping steps to increase the dispersion, deep absorption and bonding of functional materials onto the cotton fabric surface. Specifically, cotton fabrics were successively dipped into solutions containing fluorinated-decyl polyhedral oligomeric silsesquioxane (F-POSS), branched poly(ethylenimine) (PEI), and silver nanoparticles (AgNPs) of tunable colors. Through this three-step dipping and drying sequence, uniform composite films were formed on the fabric surface *via* electrostatic and chemical interactions among the coating components and cellulose fibers. The resulting textiles exhibited durable antibacterial activity, self-healing super hydrophobicity, and color tunability, with excellent resistance to washing and mechanical abrasion. The incorporation of ultrasonic assistance further improved coating uniformity, mechanical stability, and the long-term retention of AgNPs, thereby enhancing color fastness and antibacterial efficiency. Overall, this combined solution-dipping and ultrasonic-assisted technique offers a green, energy-efficient, and scalable route for developing advanced multifunctional smart fabrics suitable for protective health PPE, sportswear, and military uniforms, where long-lasting antibacterial performance, self-healing behavior, and vibrant color stability are highly desirable.^5^ Ultrasonic assisted model works upon applied in the combination of with other technology. As a research work was carried in combination of ultrasonic coating to surface grafting of fabric and develop the smart antibacterial fabric. Zwitterionic poly(3-(trimethoxysilyl) propyl methacrylate)-*co*-poly(2-(methacryloyloxy)ethyl trimethylammonium chloride) (PTMSPMA-*co*-PTMAO) copolymer were grafted onto cotton fabric (CF) to construct a stimuli-responsive surface. Sonicated surface modified fabrics were subsequently grafted with the silane groups of the copolymer and the hydroxyl groups of cellulose. This grafted coating endowed the fabric with a reversible switchable antibacterial mechanism, where the surface dynamically transitions between an “active attack” (bactericidal) mode in water and a “passive defense” (anti-adhesion) mode in sweat environments.^134^

#### 5.3.7 Plasma assisted functionalization

**Fig. 5:**
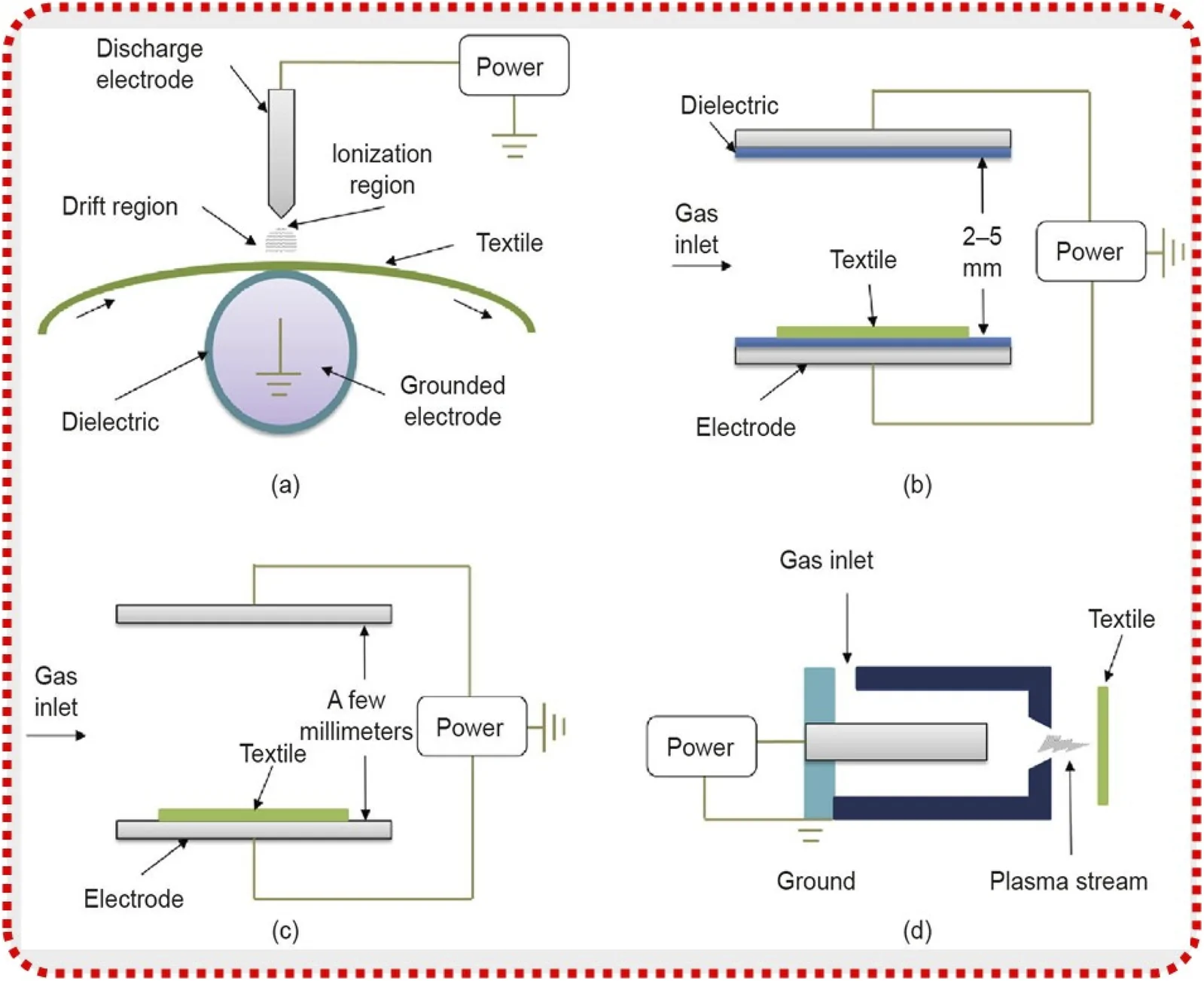
Schematic illustration of different atmospheric-pressure plasma treatments on textiles: (a) CD, (b) DBD, (c) APGD, and (d) APPJ. Redrawn with permission from [Maryam Naebe], [plasma-assisted antimicrobial finishing of textiles: a review]; published by [Elsevier], [2022].^136^

Plasma or corona treatments create reactive sites on fibers for covalent bonding of responsive polymers without altering bulk properties. This eco-friendly technique aligns with green manufacturing goals.^136^ The application of plasma in textile finishing offers a sustainable way which can markedly reduce water and energy consumption, as plasma treatments do not require conventional washing or drying steps, resulting in significant resource savings.^137^Fig. 4(b) highlights the advantages of plasma assisted antimicrobial finishing over traditional wet processes, such as the pad dry cure technique. These finishes can be obtained through polymer deposition or graft polymerization. In polymer deposition, an antimicrobial polymer is applied as a thin film onto the textile substrate, whereas graft polymerization involves covalently bonding the antimicrobial moieties directly to the fiber surface. Even when employed as a pretreatment, plasma can substantially enhance key functional properties of textiles, such as chemical uptake, while enabling comparable finishing effects with reduced water and processing time.^135^ Plasma is made up of highly concentrated reactive species which are capable modifying the physicochemical characteristics of polymer surfaces.^138^ Moreover, plasma treatment also enables multiple simultaneous surface modification processes, that could be employed to add polymer layers on fabric surface through coating or grafting. With the selection of the appropriate gases typically O_2_, H_2_, N_2_, NH_3_, air, or inert gases; and by selecting their optimizing plasma parameters such as pressure, energy, duration, and gas flow rate, the specific modifications of the textile surface can be obtained.^139^ Plasma can be at high or low temperatures; however, due to the heat sensitivity of most textile fibers, only low temperature (cold) plasma is generally used. This method could be used by direct current (DC) or alternating current (AC), with energy supplied at low frequency (1–500 kHz), radio frequency (commonly 13.56 or 27.12 MHz), or microwave frequency (typically 915 MHz or 2.45 GHz).^138^ As shown in Fig. 5, textile processing commonly employs four main types of atmospheric pressure plasma systems named as corona discharge (CD), dielectric barrier discharge (DBD), atmospheric pressure glow discharge (APGD), and atmospheric pressure plasma jets (APPJ). In a typical CD setup, the electrodes are deliberately made different from each other as one shaped like a needle and the other designed as a plate or cylinder. The plasma forms around the needle tip in what is known as the ionization region, and the active species then move toward the opposite electrode through a drift region. Due to the spacing between these electrodes are kept very small, hence, technique is especially suitable to deal with thin textile materials.^136^

Arik *et al.* observed how plasma activation (130 W, 40 s) influences the finishing performance of cotton fabrics treated with commercial antimicrobial agents containing Ag, dichlorophenol, triclosan, and diphenyl alkane derivatives, applied through a standard pad dry cure method. The pretreatment of plasma markedly enhanced both the antibacterial efficacy and the wash durability of the finishes, with the greatest improvement observed for the agents of diphenyl alkane.^140^ Plasma modification has also been shown to facilitate the adsorption and binding of chitosan onto textile fibers. In a study conducted by Naebe *et al.*,^141^ it is observed that helium and He/O_2_ atmospheric-pressure plasma treatments could promote chitosan deposition on cotton through a pad dry cure method. The plasma exposure increased surface energy and resulted in a thicker, more uniform chitosan layer. The treated fabrics demonstrated strong antimicrobial activity, particularly against the Gram-negative bacterium *Escherichia coli* (*E. coli*).^141^ Plasma treatment encompasses low pressure oxygen (800 W, 27.12 MHz, 75 Pa, 30 s) applied to viscose fabrics has been shown to enhance chitosan deposition and impart strong antibacterial properties to the treated material. Haji *et al.*^142^ demonstrated that oxygen plasma technique has enhanced the adhesion of chitosan to cotton and wool material while simultaneously increase dyeability and antimicrobial performance. In polyester (PET) material, plasma exposure increased the concentration of oxygen-containing surface groups, promoting more efficient chitosan deposition when immersed in a 2% (w/v) chitosan acetate solution followed by vacuum drying. The resulting coated textiles of chitosan exhibited high antibacterial activity against both *Escherichia coli* and *Staphylococcus aureus*. Another study produced the same outcomes when PET samples were treated with Ar/O_2_ plasma and subsequently coated with chitosan polymers and oligomers through pad bake method.^136^ A further advancement in plasma based techniques is the advancement of remote plasma assisted vacuum deposition (RPAVD), which integrates a remote plasma discharge with thermal vacuum deposition of an organic solid onto substrates, when exposed to the plasma afterglow.^143^ During this procedure, a plasma polymer matrix forms on the substrate, preserving a significant fraction of the precursor's functional groups and embedded unfragmented precursor molecules. The primary advantage of RPAVD is the fabrication of functional organic thin films with less cross-linking and higher retention of functional groups compared to customary plasma polymerization techniques. Additionally, RPAVD enables the deposition on chemically or thermally sensitive substrates at room temperature.^144^ However, the application of PDMS and other products of silicon materials is restricted by their intrinsic surface hydrophobicity, which has been associated with nonspecific protein adsorption, potentially triggering coagulation, producing immune responses, and other adverse effects during prolonged blood contact.^145^

#### 5.3.8 Advantages of smart textiles over conventional textiles

Smart antibacterial textiles offer several clear advantages over conventional antibacterial fabrics, particularly for wearable technology applications. While conventional systems rely on passive, continuous release of biocides, which often leads to rapid wash-off, reduced long-term efficacy, and environmental toxicity. Conventional antibacterial textiles rely on passive, continuous mechanisms; such as leaching of metal ions, biocidal coatings, or contact-killing surfaces; that provide immediate antimicrobial action but suffer from poor wash durability, uncontrolled release, and limited compatibility with wearable electronics. In contrast, smart antibacterial textiles operate through stimuli-responsive or self-regulated mechanisms, activating their antibacterial effect only when triggered by changes in pH, moisture, temperature, or mechanical strain. This enables controlled release, reduced environmental toxicity, and improved long-term functionality. Conventional systems are generally single-purpose, often stiffen the fabric, and degrade rapidly during laundering, whereas smart textiles integrate multiple functions, including sensing, conductivity, thermal regulation, and self-healing,while maintaining softness and breathability. For wearable technology, smart textiles clearly outperform conventional ones by offering adaptive behavior, higher durability, and better integration with flexible electronic components, making them more suitable for next-generation medical, sports, and protective applications. Conventional methods deliver strong initial antibacterial activity but poor reusability, inconsistent agent release, and potential human or ecological toxicity. Moreover, their performance, often exhibit noticeable antibacterial performance reduction after approximately 20–25 laundering cycle, failing to meet industrial durability standards for wearable textiles.^5,146^ However, increase of smart antibacterial textiles and the durability of sustained release even after several washings are noted. Table 5 is showing the comparative analysis of performance parameters between conventional and smart antibacterial textiles.

**Table 5:** Comparative analysis of performance parameters between conventional and smart antibacterial textiles

Sr. no.	Parameter	Conventional antibacterial textiles	Smart antibacterial textiles	Advantage of smart textiles	Ref.
1	Antibacterial efficiency (%)	85–99% initial reduction depending on agent	90–99% (activated/on-demand)	Comparable or higher efficiency with controlled activation	72 and 118
2	Wash durability (cycles)	Typically 15–25 cycles before significant decline	40–60+ cycles in many systems	Significantly improved long-term durability	76 and 118
3	Antibacterial mechanism	Continuous ion release or contact killing	Stimuli-responsive (pH, temperature, light, moisture, electrical)	Targeted antibacterial action reduces unnecessary exposure	5 and 103
					104 and 105
4	Release behavior	Uncontrolled, continuous leaching	Controlled or on-demand release *via* carriers	Reduced chemical loss and prolonged activity	5 and 109
5	Cytotoxicity concern	Moderate to high due to uncontrolled release (*e.g.*, Ag^+^, Cu^2+^)	Lower due to encapsulation and regulated activation	Improved biocompatibility and reduced skin irritation risk	109 and 110
6	Environmental impact	Potential nanoparticle accumulation and wastewater contamination	Reduced environmental burden through controlled release	More eco-friendly and sustainable performance	5 and 102
7	Skin microbiome compatibility	May disrupt natural microbiota due to continuous exposure	Selective activation minimizes microbiome disturbance	Better preservation of healthy skin flora	27 and 103
8	Electronic compatibility	Limited integration with conductive systems	Compatible with sensors, conductive networks, and e-textiles	Enables multifunctional wearable applications	91 and 106
9	Stimuli responsiveness	Absent	Present (pH, light, strain, moisture)	Key feature enabling adaptive functionality	5,103–105 and 115
10	Multifunctionality	Mainly antibacterial	Antibacterial + sensing + UV protection + drug delivery	Broader application potential	112
11	Manufacturing scalability	Industrially mature and cost-effective	Limited scalability for advanced systems	Opportunity for future optimization and innovation	69 and 117
12	Sustainability/recyclability	Limited recyclability due to chemical coatings	Emerging recyclable and biodegradable smart systems	Supports future circular textile development	5 and 121

### 5.4 Factors influencing the development of smart antibacterial textiles

The successful development of smart antibacterial textiles depends on several interconnected factors including antibacterial efficiency, biocompatibility, wash durability, breathability, flexibility, environmental safety, and compatibility with wearable electronic systems. Controlled release behavior, responsiveness toward external stimuli, and mechanical stability during repeated deformation are particularly important for wearable healthcare applications. In addition, long-term performance after laundering, resistance to nanoparticle leaching, and preservation of fabric comfort strongly influence practical applicability. However, several challenges continue to hinder commercialization, including cytotoxicity concerns associated with metallic nanoparticles, instability of responsive coatings under mechanical stress, limited scalability of electrospinning and microencapsulation techniques, and the absence of standardized evaluation protocols for dynamic antibacterial behavior. Future developments should therefore prioritize eco-safe multifunctional systems with improved recyclability, scalable fabrication routes, and standardized performance assessment methods.^147^

## 6 Limitations in smart antibacterial textiles

The recent research efforts aimed at advancing smart antibacterial textile technologies, which are a key driver of market expansion. Although the development of this sector is in early stages, yet the potential for innovative smart fabrics to significantly enhance the performance and versatility of conventional textiles is substantial.^148^ Current market trend for Medical Smart Textile Market indicates that a compound annual growth rate (CAGR) of 7.51%, with the sector expected to reach USD 2.10 billion by 2027 growing estimative of 7.51%, reaching USD 2.10 billion by the end of 2027. The pursuit of improved healthcare solutions demands both the current scenario and in the long-term scalability, which are closely linked to the progress in implant of textile materials, such as artificial ligaments and tendons. Another important aspect is to enhance global life expectancy, which is anticipated to raise the number of surgical procedures and, consequently, the demand for implantable and medical-grade textile products. Woven, knitted, and nonwoven materials are forecasted to experience imminent growth between 2017 and 2028, mainly because hygiene related items account for nearly half of the global medical textile market. A recent and the most catastrophic health incident is COVID-19, which illustrated the scale of investment in this sector, prompting substantial procurement of hospital beds and equipment to manage more than 36 million cases. It also compels to improve the domestic manufacturing capacity and personal protective equipment production. Additional product development includes both biodegradable and non-biodegradable polymer items, such as face masks, shoe covers, and maternity or sanitary pads.^148^ Smart systems consistently maintain higher antibacterial performance after multiple wash cycles and demonstrate added functionalities such as sensing, UV shielding, or thermal response. Nonetheless, few studies assess *cytotoxicity* or *ecological leaching* under real laundering conditions an urgent area for standardization. Many smart systems rely on metal nanoparticles, conductive polymers, or photocatalytic oxides, which may generate reactive oxygen species (ROS) or release ions that pose cytotoxic risks to human skin or surrounding tissues.^16,17^ Despite improved structural stability, very few studies evaluate their behavior under *real laundering conditions*, especially concerning ecological leaching, nanoparticle detachment, or long-term dermal exposure, areas that require urgent standardization. In addition, advanced fabrication techniques such as electrospinning, sol–gel deposition, and microencapsulation offer precise control over stimuli-responsive release, but they remain resource-intensive, time-consuming, and unsuitable for industrial-scale throughout.^133^ Conductive components such as graphene, CNTs, and metallic nanostructures enhance sensing and self-regulation, yet their integration introduces challenges in maintaining fabric softness, flexibility, and wearer comfort—issues especially relevant for wearable electronics.^49–52^ Environmentally, smart systems reduce uncontrolled leaching but raise new questions about recyclability and end-of-life processing, particularly when non-biodegradable nanomaterials or synthetic polymers are involved.^41^ Overall, the translation of smart antibacterial textiles from laboratory research to commercial wearable devices remains constrained by scalability, cost, toxicological uncertainty, and the absence of standardized evaluation methods. Current ISO and AATCC antibacterial standards measure static inhibition or zone-of-clearance and do not account for real-time activation, transient responses, or pH/moisture-triggered release mechanisms. Smart materials that rely on photocatalysis, ROS generation, or ionic activation require *dynamic* evaluation, yet no formal standards exist to capture these mechanisms.^133^ Second most important limitation is related to long term compatibility. As many smart antibacterial systems rely on metal/metal-oxide nanoparticles (Ag, CuO, TiO_2_, ZnO), carbon nanostructures (CNTs, graphene), or conductive polymers whose long-term dermal compatibility is still uncertain; because ions can induce oxidative stress and increased intracellular ROS, potentially causing protein, lipid, and DNA damage.^16^ Existing research studies rarely assess chronic skin exposure, nanoparticle penetration, or dermal absorption under sweat or mechanical stress, making toxicological profiling a major research necessity.^30,36^ Another, limitation is to provide the energy and difficulties during sensor integration. Responsive textiles capable of sensing, actuation, or thermal/chemical feedback require stable energy input. While triboelectric and piezoelectric systems offer self-powered options, they often add structural complexity, stiffness, or increased fabrication cost. Integrating conductive agents such as CNTs, graphene, and metal nanostructures can compromise comfort or cause hotspots due to localized charge buildup.^51,52^ Third most important issue belongs to the scalability and production cost. Many fabrication techniques used to produce smart antibacterial textiles as already described in aforementioned sections such as electrospinning, microencapsulation, plasma activation, sol–gel, layer-by-layer functionalization are expensive, slow, or unsuitable for continuous large-scale textile processing. Electrospinning requires high energy input and limited production width; microencapsulation is chemically intensive; and plasma systems require specialized vacuum or atmospheric equipment, restricting industrial adoption.^78,141^ Futhermore, smart systems reduce uncontrolled leaching compared to conventional finishes, long-term environmental risks such as nanoparticle release, microplastic shedding, and non-biodegradable polymer matrices remain understudied. Plant-based or biodegradable agents provide eco-friendly alternatives, yet their stability and wash-durability are still limited.^29^ End-of-life recycling or safe degradation pathways for textiles containing metal nanoparticles, conductive carbon materials, or hybrid polymer–nanomaterial composites are also rarely explored. Even after, promising laboratory results, these smart mechanisms are often tested only under idealized conditions. Long-term field data on mechanical stress, perspiration, or laundering remain scarce, representing a significant research gap.

## 7 Cytotoxicity, biocompatibility, skin microbiome, and standardization challenges

### 7.1 Cytotoxicity and biocompatibility of conventional antibacterial agents

Although conventional antibacterial textiles provide effective protection against microbial contamination, concerns regarding cytotoxicity and long-term biocompatibility remain significant barriers to their widespread adoption in healthcare and wearable applications. The toxicity profile of antibacterial textiles depends largely on the type, concentration, release behavior, and exposure duration of the incorporated antimicrobial agents. Silver nanoparticles (AgNPs), among the most extensively used antibacterial agents in textiles, exhibit broad-spectrum antimicrobial activity through ion release and reactive oxygen species (ROS) generation. However, several *in vitro* studies have demonstrated concentration-dependent cytotoxic effects on human keratinocytes and dermal fibroblasts. Excessive Ag^+^ release may induce oxidative stress, mitochondrial dysfunction, DNA damage, and inflammatory responses in skin cells. While low concentrations generally remain within acceptable safety limits, prolonged exposure and nanoparticle leaching raise concerns regarding chronic skin contact and environmental accumulation.^149^ Copper oxide (CuO) nanoparticles exhibit similar antibacterial mechanisms through ROS generation and membrane disruption. However, elevated copper ion concentrations have been reported to induce cellular oxidative stress and inflammatory responses in human epithelial cells. Likewise, ZnO nanoparticles, although generally regarded as biocompatible at moderate concentrations, may cause cytotoxic effects through excessive Zn^2+^ ion release and intracellular ROS production when present at high loading levels.^150^ Carbon-based nanomaterials such as carbon nanotubes (CNTs) and graphene derivatives offer strong antibacterial performance through membrane piercing and electron-transfer mechanisms. Nevertheless, concerns remain regarding their potential to induce oxidative stress, inflammatory signaling pathways, and cellular membrane damage. Surface functionalization and immobilization strategies have been proposed to reduce these risks and improve compatibility with skin-contact applications.^151^ Natural antibacterial agents including chitosan, alginate, sericin, plant polyphenols, and essential oils generally demonstrate superior biocompatibility compared with metal-based systems. Chitosan-based coatings, in particular, have shown excellent cytocompatibility with fibroblasts and keratinocytes while simultaneously promoting wound healing and tissue regeneration. However, variability in botanical extract composition and potential allergenic reactions must still be considered during product development. Consequently, future antibacterial textile development should focus on minimizing uncontrolled agent release, reducing nanoparticle leaching, and conducting comprehensive biological evaluations including ISO 10993-based cytotoxicity, irritation, sensitization, and long-term skin-contact assessments before clinical implementation.^152^

### 7.2 Cytotoxicity and biocompatibility of smart antibacterial systems

Smart antibacterial textiles have been proposed as a safer alternative to conventional continuously active antimicrobial systems because they enable controlled, stimuli-responsive activation. By releasing antimicrobial agents only in response to specific triggers such as pH variation, moisture accumulation, temperature change, bacterial metabolites, or external electrical stimulation, these systems may significantly reduce unnecessary exposure of human tissues to antimicrobial compounds.^5^ Hydrogel-based antibacterial textiles fabricated from poly(*N*-isopropylacrylamide), chitosan, alginate, and other biocompatible polymers generally demonstrate favorable cytocompatibility due to their high water content and structural similarity to extracellular matrices. Several studies have reported excellent viability of fibroblasts and keratinocytes when exposed to these materials, supporting their suitability for wound healing and wearable biomedical applications.^153^ Microencapsulation technologies further improve safety by physically isolating antimicrobial agents within polymeric carriers and regulating their release kinetics. This approach reduces burst release phenomena commonly observed in conventional coatings and minimizes local cytotoxicity associated with high concentrations of silver ions, antibiotics, or biocides.^154^ Nevertheless, smart systems are not inherently risk-free. Conductive nanomaterials such as graphene, MXenes, carbon nanotubes, and metallic nanowires integrated into wearable sensors may produce oxidative stress, inflammatory responses, or cellular membrane damage depending on particle size, concentration, and degree of exposure. Furthermore, repeated mechanical deformation during textile use may accelerate nanoparticle release, thereby altering toxicological profiles over time.^155^

### 7.3 Impact on skin microbiome of smart antibacterial systems

The human skin hosts a diverse microbiome that contributes to immune regulation, pathogen resistance, wound healing, and maintenance of skin barrier integrity. While antibacterial textiles are designed to suppress pathogenic microorganisms, excessive or non-selective antimicrobial activity may unintentionally disrupt beneficial microbial communities.^156^ Conventional antibacterial textiles often rely on continuous release of antimicrobial agents, which can indiscriminately affect both harmful and commensal microorganisms. Long-term exposure to silver ions, quaternary ammonium compounds, triclosan, and other broad-spectrum antimicrobials has been associated with alterations in microbial diversity and ecological imbalance. Such disturbances may increase susceptibility to opportunistic infections, skin irritation, or dysbiosis.^157^

### 7.4 Standard evaluation methods for antibacterial smart textiles and standardization gaps

The antibacterial performance of textile materials is commonly evaluated using standardized protocols such as ISO 20743, AATCC 100, ASTM E2149, and JIS L 1902. ISO 20743 and AATCC 100 are among the most widely adopted methods for determining quantitative bacterial reduction on textile surfaces under controlled laboratory conditions. However, these conventional protocols were primarily developed for passive antibacterial systems and often fail to capture the dynamic behavior of stimuli-responsive smart textiles. For example, smart antibacterial fabrics activated by pH, temperature, light, or mechanical deformation may exhibit delayed or condition-dependent antimicrobial responses that are not adequately represented in static incubation environments. Similarly, conventional standards rarely evaluate repeated activation cycles, long-term responsiveness, or compatibility with wearable electronic components. Therefore, future testing protocols should incorporate simulated sweat environments, cyclic mechanical deformation, temperature-responsive activation, dynamic moisture exposure, and real-time monitoring conditions to better evaluate multifunctional smart antibacterial systems.^157–159^

Despite rapid technological advances, standardized biocompatibility assessment protocols specifically designed for stimuli-responsive antibacterial textiles remain limited. Most studies evaluate antibacterial efficacy while neglecting long-term cytotoxicity, repeated skin-contact exposure, immunological responses, and aging-related release behavior. Future research should therefore combine antibacterial performance testing with comprehensive biological evaluations under realistic wearable conditions to establish clinically relevant safety profiles.^155^ Smart antibacterial textiles offer a potentially improved strategy because antimicrobial activity is activated only under specific environmental conditions. Controlled and localized activation may reduce unnecessary exposure of beneficial microorganisms while maintaining protection against pathogenic species. However, very few studies have systematically investigated microbiome preservation following prolonged use of smart antibacterial fabrics, representing a major knowledge gap in the field.^160^ Another critical challenge is the absence of standardized testing methods capable of evaluating dynamic antibacterial responses. Current textile standards such as ISO 20743, AATCC 100, ASTM E2149, and JIS L 1902 were primarily developed for passive antibacterial systems and focus on bacterial reduction under static laboratory conditions. These protocols do not account for trigger-dependent activation, controlled release kinetics, repeated stimulation cycles, sensor–actuator interactions, or multifunctional wearable behavior.^158^

Future standardization efforts should incorporate:

(i) Trigger-responsive antibacterial testing under simulated physiological conditions;

(ii) Repeated activation–deactivation cycling;

(iii) Wash durability combined with activation efficiency;

(iv) Skin-cell cytotoxicity assessments according to ISO 10993 guidelines;

(v) Microbiome preservation studies; and

(vi) Integrated evaluation of sensing, antimicrobial, and wearable performance.

Development of such comprehensive standards will be essential for the successful translation of smart antibacterial textiles from laboratory-scale demonstrations to clinically safe and commercially viable wearable technologies.^157^ A summary of the most widely adopted antibacterial testing standards and their limitations for smart antibacterial textiles is presented in Table 6.

**Table 6:** Existing standards and their limitations for smart antibacterial textiles

Sr. no	Standard	Purpose	Suitable for conventional textiles	Limitation for smart textiles	Ref.
1	ISO 20743	Quantitative antibacterial activity	Yes	Cannot assess stimuli-triggered activation	158
2	AATCC 100	Bacterial reduction measurement	Yes	No evaluation of controlled release behavior	159
3	ASTM E2149	Dynamic contact antibacterial testing	Yes	Does not simulate wearable physiological conditions	161
4	JIS L 1902	Antibacterial efficacy assessment	Yes	Assumes continuous antibacterial action	162
5	ISO 10993	Biocompatibility and cytotoxicity	Partially	Does not address microbiome effects or smart-textile activation cycles	163

## 8 Conclusion

The development of antibacterial textiles has progressed from conventional passive antimicrobial finishes to advanced smart textile systems capable of responding to environmental and physiological stimuli. Conventional antibacterial textiles remain widely used due to their simple fabrication routes and broad-spectrum antimicrobial activity. However, their performance is often constrained by uncontrolled biocide release, limited wash durability, declining long-term efficacy, and concerns regarding chemical leaching, cytotoxicity, environmental contamination, and disruption of the natural skin microbiome. These limitations have driven the search for innovative, biocompatible, and controlled antimicrobial delivery systems. Recent advances in smart antibacterial textiles have introduced stimuli-responsive platforms that enable on-demand antimicrobial activation and controlled therapeutic agent release. These systems employ advanced functional materials, including pH-responsive hydrogels, electrothermally conductive polymers, microencapsulated antimicrobial agents, graphene-based nanomaterials, nanofibrous networks, and biohybrid multifunctional composites that respond to specific external or physiological triggers. Compared with conventional antibacterial finishes, these smart systems offer improved durability, targeted antimicrobial action, reduced unnecessary biocide exposure, and opportunities for integrating sensing, energy-harvesting, self-monitoring, and wearable electronic functionalities. This review comprehensively summarized the major classes of antibacterial agents, their mechanisms of action, and their implementation in both conventional and smart antibacterial textiles. In addition, fabrication strategies, durability, wash stability, sustainability, and current therapeutic approaches, including biohybrid and bacteria-assisted responsive systems, were critically evaluated. Although traditional finishing techniques, such as pad–dry–cure and sol–gel processing, continue to provide effective antimicrobial performance, their long-term functionality remains limited by poor durability and uncontrolled release behavior. Overall, smart antibacterial textiles represent a significant advancement toward multifunctional, durable, and adaptive wearable healthcare systems, although further improvements are required before their widespread clinical and commercial implementation.

## 9 Future outlook

Future research should focus on improving the long-term durability, biocompatibility, scalability, and sustainability of smart antibacterial textiles while facilitating their translation into practical wearable healthcare applications. The development of multifunctional textile platforms integrating controlled antimicrobial delivery, biosensing, energy harvesting, self-powered operation, and wireless communication is expected to drive the next generation of wearable medical systems. A key priority is the establishment of standardized testing protocols specifically designed for stimuli-responsive antibacterial textiles. Current antibacterial textile standards primarily assess static antimicrobial performance and therefore do not adequately evaluate smart systems operating under dynamic physiological conditions. Future evaluation protocols should include pH-triggered antibacterial activation under simulated sweat conditions (pH 4–7), temperature-responsive testing (25–40 °C), cyclic bending and mechanical strain analysis, laundering durability beyond 50 wash cycles, and time-dependent antibacterial activation measurements. Adapting existing standards to these realistic wearable conditions will enable more reliable performance assessment and facilitate regulatory acceptance.^6,157^ In addition, the end-of-life management of multifunctional smart antibacterial textiles remains a significant challenge because the integration of metallic nanoparticles, conductive coatings, polymers, and electronic components complicates recycling and material recovery. Future efforts should therefore emphasize biodegradable antimicrobial carriers, recyclable conductive materials, green synthesis routes, and disassembly-friendly textile architectures to improve the environmental sustainability and circularity of wearable e-textiles.^160^ Overall, continued interdisciplinary collaboration among materials scientists, textile engineers, microbiologists, biomedical researchers, and industry will be essential to accelerate the commercialization of safe, durable, and sustainable smart antibacterial textiles for advanced healthcare and wearable electronic applications.

## Author contributions

Azam Ali: conceptualization, methodology, literature investigation, data curation, analysis and interpretation of the literature, visualization, writing – original draft, writing – review and editing, supervision, project administration, and correspondence with the journal. Muhammad Zaman Khan: literature investigation, data collection, analysis of antibacterial textile technologies, and writing – review and editing. Jiri Militký: supervision, critical evaluation of textile materials and antibacterial technologies, and writing – review and editing. Jakub Wiener: literature investigation, technical analysis of textile fabrication and functionalization approaches, and writing – review and editing. Tazeen Rana: literature investigation, analysis of smart antibacterial and biomedical textile applications, and writing – review and editing. Eman Kashita: literature investigation, data curation, analysis of antibacterial mechanisms and smart textile systems, and writing – review and editing. All authors contributed to the interpretation of the literature, critically reviewed the manuscript, and approved the final version.

## Conflicts of interest

The authors declare that there are no conflicts of interest related to this work.

## Data Availability

No new data were generated or analysed in this study.
